# MIF-CD74 signaling drives immune modulation in medulloblastoma

**DOI:** 10.1093/neuonc/noag020

**Published:** 2026-02-06

**Authors:** Benjamin Draper, Zhen You, Dean Thompson, Xu Guo, Alaide Morcavallo, Diego Chillon Pino, Carlos Lorenzo Gido Nery, Sumana Shrestha, Chantelle E Bowers, Courtney Himsworth, Alberto Delaidelli, Bethany Remeniuk, Sonia Morlando, Brandon Wade, Freya Gordon, Yara Sanchez-Corrales, Bei Hopkins, Natalie Monteiro, Darren Locke, Miao Liu, Jacob Torrejon Diaz, Kevin Greenslade, Barbara Martins da Costa, Karen Barker, Colin Kwok, Olumide Ogunbiyi, Anya Fletcher, Stacey Richardson, Carlos Custodia, Rafael Roque, Thomas Jackson, Regan Barfoot, Sergi Castellano, Rebecca M Hill, Olivier Saulnier, Thomas S Jacques, Michael D Taylor, Claudia C Faria, Olivier Ayrault, Poul H Sorensen, John Anderson, Louis Chesler, L Frank Huang, Steven C Clifford, Laura K Donovan

**Affiliations:** UCL Great Ormond Street Institute of Child Health, London, UK; Department of Pediatric and Adolescent Medicine, Department of Biochemistry and Molecular Biology, Mayo Clinic Comprehensive Cancer Center, Mayo Clinic College of Medicine and Science, Rochester, Minnesota, USA; Wolfson Childhood Cancer Research Centre, Newcastle University Centre for Cancer, Translational and Clinical Research Institute, Newcastle University, Newcastle Upon Tyne, UK; Department of Pediatric and Adolescent Medicine, Department of Biochemistry and Molecular Biology, Mayo Clinic Comprehensive Cancer Center, Mayo Clinic College of Medicine and Science, Rochester, Minnesota, USA; Centre for Paediatric Oncology Experimental Medicine, Institute of Cancer Research, Sutton, UK; UCL Great Ormond Street Institute of Child Health, London, UK; UCL Great Ormond Street Institute of Child Health, London, UK; UCL Great Ormond Street Institute of Child Health, London, UK; Centre for Paediatric Oncology Experimental Medicine, Institute of Cancer Research, Sutton, UK; UCL Great Ormond Street Institute of Child Health, London, UK; UCL Great Ormond Street Institute of Child Health, London, UK; BC Cancer Centre, Vancouver, Canada; Department of Pathology, Mass General Brigham, Harvard Medical School, Boston, Massachusetts, USA; Akoya Biosciences, Marlborough, Massachusetts, USA; UCL Great Ormond Street Institute of Child Health, London, UK; UCL Great Ormond Street Institute of Child Health, London, UK; UCL Great Ormond Street Institute of Child Health, London, UK; UCL Great Ormond Street Institute of Child Health, London, UK; Akoya Biosciences, Marlborough, Massachusetts, USA; Akoya Biosciences, Marlborough, Massachusetts, USA; Akoya Biosciences, Marlborough, Massachusetts, USA; Department of Pediatric and Adolescent Medicine, Department of Biochemistry and Molecular Biology, Mayo Clinic Comprehensive Cancer Center, Mayo Clinic College of Medicine and Science, Rochester, Minnesota, USA; Institut Curie, PSL Research University, Université Paris Sud, Université Paris-Saclay, CNRS, Paris, France; Centre for Paediatric Oncology Experimental Medicine, Institute of Cancer Research, Sutton, UK; Centre for Paediatric Oncology Experimental Medicine, Institute of Cancer Research, Sutton, UK; Centre for Paediatric Oncology Experimental Medicine, Institute of Cancer Research, Sutton, UK; Centre for Paediatric Oncology Experimental Medicine, Institute of Cancer Research, Sutton, UK; UCL Great Ormond Street Institute of Child Health, London, UK; Wolfson Childhood Cancer Research Centre, Newcastle University Centre for Cancer, Translational and Clinical Research Institute, Newcastle University, Newcastle Upon Tyne, UK; Wolfson Childhood Cancer Research Centre, Newcastle University Centre for Cancer, Translational and Clinical Research Institute, Newcastle University, Newcastle Upon Tyne, UK; Gulbenkian Institute for Molecular Medicine (GIMM), Lisboa, Portugal; Neurosurgery Department, Hospital de Santa Maria, Unidade Local de Saúde de Santa Maria (ULSSM), Lisboa, Portugal; Clínica Universitária de Neurocirurgia, Faculdade de Medicina da Universidade de Lisboa, Lisboa, Portugal; Gulbenkian Institute for Molecular Medicine (GIMM), Lisboa, Portugal; Neurosurgery Department, Hospital de Santa Maria, Unidade Local de Saúde de Santa Maria (ULSSM), Lisboa, Portugal; Clínica Universitária de Neurocirurgia, Faculdade de Medicina da Universidade de Lisboa, Lisboa, Portugal; UCL Great Ormond Street Institute of Child Health, London, UK; Centre for Paediatric Oncology Experimental Medicine, Institute of Cancer Research, Sutton, UK; UCL Great Ormond Street Institute of Child Health, London, UK; Wolfson Childhood Cancer Research Centre, Newcastle University Centre for Cancer, Translational and Clinical Research Institute, Newcastle University, Newcastle Upon Tyne, UK; Inserm U1330, Genomics and Development of Childhood Cancers Lab, Institut Curie, PSL University, SIREDO Oncology Center, Paris, France; UCL Great Ormond Street Institute of Child Health, London, UK; Great Ormond Street Children’s Hospital, London, UK; Texas Children’s Cancer and Hematology Center, Houston, Texas, USA; Department of Pediatrics—Hematology/Oncology, Baylor College of Medicine, Houston, Texas, USA; Department of Neurosurgery, Baylor College of Medicine, Houston, Texas, USA; Department of Neurosurgery, Texas Children’s Hospital, Houston, Texas, USA; Gulbenkian Institute for Molecular Medicine (GIMM), Lisboa, Portugal; Neurosurgery Department, Hospital de Santa Maria, Unidade Local de Saúde de Santa Maria (ULSSM), Lisboa, Portugal; Clínica Universitária de Neurocirurgia, Faculdade de Medicina da Universidade de Lisboa, Lisboa, Portugal; Institut Curie, PSL Research University, Université Paris Sud, Université Paris-Saclay, CNRS, Paris, France; BC Cancer Centre, Vancouver, Canada; UCL Great Ormond Street Institute of Child Health, London, UK; Great Ormond Street Children’s Hospital, London, UK; Centre for Paediatric Oncology Experimental Medicine, Institute of Cancer Research, Sutton, UK; Department of Pediatric and Adolescent Medicine, Department of Biochemistry and Molecular Biology, Mayo Clinic Comprehensive Cancer Center, Mayo Clinic College of Medicine and Science, Rochester, Minnesota, USA; Wolfson Childhood Cancer Research Centre, Newcastle University Centre for Cancer, Translational and Clinical Research Institute, Newcastle University, Newcastle Upon Tyne, UK; UCL Great Ormond Street Institute of Child Health, London, UK

**Keywords:** CD74, immune-suppression, macrophages, relapsed medulloblastoma, tumor-immune microenvironment

## Abstract

**Background:**

Relapsed medulloblastoma remains a significant therapeutic challenge as it is near universally fatal. The tumor microenvironment of medulloblastoma plays a critical role in tumor progression, influencing tumor growth, immune evasion, and therapeutic resistance. We hypothesized that defining tumor-immune interactions in diagnostic and relapsed medulloblastoma may uncover mechanisms of immune evasion and identify novel therapeutic targets.

**Methods:**

We analyzed paired primary and recurrent RNA-sequencing data from 140 medulloblastoma patients to profile immune cell composition and validate spatial relationships within the TME. To identify key tumor-immune interactions, we developed a novel algorithm to detect receptor-ligand pairs using single-cell RNA-sequencing data. These interactions were validated across RNA and proteomic datasets. Their functional significance was empirically demonstrated in newly developed immunocompetent models of recurrent medulloblastoma that closely recapitulate the human disease.

**Results:**

We observed a shift toward a heightened immunosuppressive TME at relapse. Using our algorithm, we identified biologically significant receptor-ligand interactions, most notably MIF-CD74, constitutively expressed at RNA and protein levels across medulloblastoma subgroups, at diagnosis and relapse. Disrupting MIF-CD74 interactions led to significant alterations in the tumor microenvironment, highlighting its functional significance.

**Conclusions:**

Our multifaceted approach identified key tumor-immune interactions in medulloblastoma. Among these, MIF-CD74 was validated as a targetable interaction, demonstrating the utility of our integrative approach for identifying novel therapeutic targets across multiple tumor types.

## Graphical Abstract

**Graphical Abstract:**
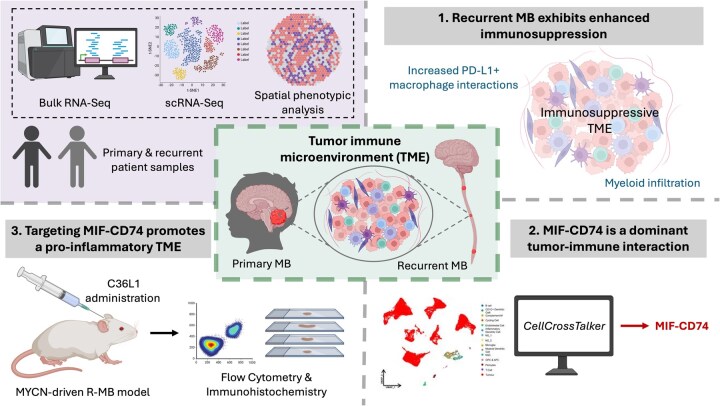

Key Points

- Transcriptomics and spatial profiling reveal relapsed medulloblastoma exhibits enhanced immunosuppression.
- MIF-CD74 signaling is a dominant tumor–immune interaction.
- Targeting MIF-CD74 promotes a pro-inflammatory tumor microenvironment.

Importance of the Study

Relapsed medulloblastoma remains a major clinical challenge, with poor survival outcomes and limited therapeutic options. While the tumor microenvironment has emerged as a critical determinant of tumor progression and therapeutic resistance in various cancers, its role in medulloblastoma, particularly at relapse, remains poorly defined. This study provides the first comprehensive spatial and transcriptomic characterization of the immune landscape in Group3 and Group4 medulloblastoma at diagnosis and recurrence. We identify a shift toward an immunosuppressive TME at relapse, marked by sustained myeloid infiltration and increased PD-L1^+^ macrophage interactions. Using a novel computational pipeline, *CellCrossTalker*, we uncover the MIF-CD74 axis as a key mediator of immune suppression and validate its therapeutic potential in an immune-competent MYCN-driven relapsed medulloblastoma model. These findings highlight the immune evasion mechanisms underpinning medulloblastoma recurrence and offer a rational framework for targeting the tumor microenvironment to improve immunotherapeutic efficacy in high-risk medulloblastoma.

One of the most significant unmet clinical challenges in pediatric oncology is the development of novel therapies for relapsed medulloblastoma (R-MB). Following conventional upfront cranio-spinal irradiation (CSI)-based treatments, MB relapse occurs in around 30% of cases and is near universally fatal, accounting for 10% of all childhood cancer deaths.^1^ Integrative genomic analyses have elucidated that MB comprises four distinct principal molecular subgroups (WNT, SHH, Group3, Group4), each characterized by unique clinical and molecular attributes.^2^

The tumor microenvironment (TME) is as a crucial regulator of tumor progression, influencing tumor growth, immune evasion, and therapeutic resistance.^3^ Tumor-infiltrating immune cells have shown prognostic relevance and predictive value for chemotherapeutic response in various solid tumors.^4^^,^^5^ Emerging research indicates that MBs may harbor fewer immune cells compared to glioblastomas,^6^^,^^7^ which are notoriously recognized as an immunological desert with few to virtually absent infiltrating immune effector cells.^8^ Despite extensive molecular characterization of MB, the use of immunotherapies remains limited, likely due to insufficient understanding of the TME and any prognostically relevant immune cells that contribute to it.^6^^,^^9^^,^^10^ A limited number of studies have shown that MB subgroups harbor few infiltrating immune cells, with SHH-MB exhibiting an immune signature characterized by the presence of fibroblasts, T-cells, and macrophages, while enrichment of markers associated with cytotoxic lymphocytes and myeloid dendritic cells have been reported in Group3 and Group4-MB tumors.^6^^,^^9^

The tumor immune microenvironment (TIME) in R-MB remains poorly understood.^11^^,^^12^ Although significant progress has been made in understanding the TME at diagnosis, little is known about the composition and interactions of immune cells within relapsed disease and their potential roles in driving tumor progression, immune evasion, and treatment resistance.^10^ Substantial genetic alterations accumulate during disease progression, with more than 40% of the genomic landscape diverging in R-MB.^13^ Understanding the interaction between the TIME, tumor-intrinsic factors, and cellular composition is crucial for developing targeted interventions^14^ that disrupt pathways involved in tumor development, progression, and recurrence.^12^

We hypothesized that deciphering the MB TIME composition and its key cellular communications could reveal dominant immune-suppressive mechanisms, identifying critical vulnerabilities for therapeutic exploitation. In this study, we employ genomic, spatial, and phenotypic investigative approaches to characterize the immune composition of Group3 and Group4-MB at diagnosis and relapse. We report a novel algorithm pipeline for the identification of crucial cellular interactions within the TME and demonstrate its potential to reveal therapeutic vulnerabilities using a newly characterized MYCN-driven model of R-MB.

## Methods

### Multiplexing PhenoImager From Akoya Biosciences - Spatial Phenotypic Analysis

A 6-plex, 7-color mIF panel was optimized for formalin-fixed paraffin-embedded (FFPE) pediatric MB tissues. Analytical performance followed published guidelines.^15^

A Leica Bond-RX autostainer protocol for Akoya’s MOTiF PD-1/PD-L1 Panel kit (CD3 replaced with CD8) was adapted for FFPE pediatric MB. Staining order and libraries are outlined in Supplementary Methods.^15^

Akoya’s R-packages, phenoptr and phenoptrReports, reduced high-dimensional single-cell data (cell location/counts/density/marker intensity) into staining statistics, ­tissue regions, and spatial parameters to identify two ­phenotypes of interest.

### Protein Synthesis

All peptides were synthesized by Proteogenix (Schiltigheim, France) with an NH2 C-terminal modification. The sequences of the peptides are C36L1: KSSQSVFYSSNNKNYLA, Scrambled: SNKVNLSSSNKFYQSYA.

### In Vivo Models - UCL

All studies were UK Home Office approved (PPL: PP5675666). FVB/Nrj mice (Janvier Labs, France) were bred in-house and housed in vented cages (max 5/cage).

For Glt1-tTA (glutamate transporter 1-tetracycline transactivator) and tetracycline response element (TRE)-MYCN/luciferase (Luc) (Glt1-tTA/TRE-MYCN-Luc; GTML) allografts, 2 × 10^5^ cells in 3 μL were injected into the cerebellum (coordinates: *x* = 1 mm, *y* = –2 mm) using a Hamilton syringe as described previously.^16^ Mice were monitored weekly via bioluminescent imaging (BLI) after subcutaneous injection of 15 mg/kg d-Luciferin, followed by anesthesia and imaging on IVIS Lumina-III (10 000 photon threshold, 300 s max).

Mice were randomized using CARMM^17^ with weight and sex as covariates. For short-term therapy studies, C36L1 peptide (1 mg/kg) was injected intraventricularly on days 0 and 8; 10 mg/kg was given intraperitoneally daily from day-3. Controls received vehicle or scrambled peptide. Imaging was done on days 7 and 14. On day-14, mice were sacrificed, and tumors/cerebellum collected. For long-term survival studies, C36L1 peptide (1 mg/kg) or scrambled peptide were administered intraventricularly when tumors reached 1 × 10^5^ photons/second. Treatments were continued once weekly for a further two weeks and animals were imaged bi-weekly until humane endpoint. Tumors were filtered, lysed, pelleted (300 rcf, 10 min), resuspended in PBS, and 1 × 10^6^ cells used for flow cytometry.

### In Vivo Models - Institute of Cancer Research

All procedures were approved by the ICR Animal Welfare and Ethical Review Body, following UK Home Office and NCRI cancer research guidelines. Glt1-tTA/TRE-MYCN-Luc mice were bred on an FVB/NJ background, with ad libitum food/water access and humane euthanasia.

Following BLI, animals with signals >1 × 10^8^ photons/sec were split into control and radiation groups. Weekly imaging continued until signs of tumor-related intracranial pressure prompted euthanasia.

Craniospinal irradiation was delivered using CT-guided SARRP (XStrahl) with a variable collimator. Under anesthesia, mice were positioned on a heated bed. CT scans guided arc treatment with 3 isocenters: 1 brain field (–90°, 90°) and 2 spine fields (–45°, 45°). Mice received 3 Gy (brain) and 2 Gy (spine) over a 5-on/2-off schedule, totaling 54 Gy, 36 Gy, or modulated doses (12 Gy, 8 Gy; α/β = 10). Body weight was tracked daily during treatment and biweekly post-treatment to assess tolerability.

### Flow Cytometry

Flow cytometry staining was performed in 96 well plates (100 μL). Data, including fluorescence minus-one (FMO and compensation controls, were acquired using a FACSymphony A5 with FACSDiva (BD). Compensation was calculated and data were analyzed using FlowJo (TreeStar). Multi-parametric flow cytometry data was analyzed using a python implementation of FlowSOM^18^ to cluster samples. Antibodies and identifications are listed in Supplementary Methods.

### Immunohistochemistry (IHC) and Histology-Scores

Formalin-fixed, paraffin-embedded sections were ­analyzed for CD74 expression. In brief, tissue sections were incubated in Tris-EDTA buffer (cell conditioning 1; CC1) at 95 ˚C for 1-h to retrieve antigenicity, followed by incubation with CD74 antibody (Origene CF507339) at 1:500 for 1-h. Slides were then incubated with secondary antibody (Jackson Laboratories) with 1:500 dilution followed by Ultramap HRP and Chromomap DAB detection. Intensity scoring was done on a common four-point scale.^19^

### Patient Samples and RNA-Sequencing Data Processing - Human

A total of 140 samples, taken from patients at diagnosis and/or relapse were used in this analysis: 86 were obtained from a previous study with pre-processed RNA libraries;^10^ 54 samples were prepared using either RNA-Sequencing (*n* = 19), or RNA-Capture (*n* = 35) library kits, using the TruSeq RNA Library Prep and Agilent SureSelect xThs RNA+v8 Exome Capture Kit respectively.

Quality was assessed with FastQC, aligned to hg19 using RNA-STAR,^20^ and gene counts generated via HT-seq count. Counts were merged into a single matrix in R (v4.3.2 “Eye Holes”). Data were normalized using DESeq2 packages: variance stabilizing transformation (vst), batch-corrected with ComBat (sva),^21^ and converted to TPM for deconvolution.

### Deconvolution Analysis - Human

To deconvolve MB tumors, a high-resolution signature was built by combining LM22 immune data^22^^,^^23^ with RNA-array data from MB cell lines (*n* = 8), astrocytes (*n* = 3, E-MTAB-4771), brain endothelial cells (*n* = 4, GSE12679), and neurons (*n* = 6, GSE12679). Data were normalized using normalizeBetweenArrays (Limma package)^24^ and visualized via uniform manifold approximation and projection (UMAP).

CIBERSORTx^25^ was used to generate the signature matrix. Patient TPM data were applied using CIBERSORTx in B-mode (quantile normalization off, 1000 permutations) to estimate cell-type fractions. Results were analyzed in R.

Immune cell proportions were compared across molecular groups (SHH, Group3, Group4) using one-way ANOVA, and between diagnosis vs. recurrence using *t*-tests (ggpubr). Visualizations were created with ggplot2.

### RNA-Sequencing Data Processing - Mouse

Murine RNA was sequenced externally. Raw fastq files were quality-checked with FastQC, aligned to mm39 using RNA-STAR, and gene counts generated via HT-seq count. Counts were loaded into R (DESeq2) and normalized to TPM for deconvolution.

To compare mouse and human MB profiles, data were variance-stabilized, annotated with gene names/functions using BiomaRt,^26^ and filtered for protein-coding genes with human orthologs. These were combined with vst-transformed human diagnostic data and visualized using UMAP.

### Deconvolution Analysis - Mouse

A deconvolution signature was built using RNA-Seq data from purified murine immune cells (*n* = 205, GSE109125) and MB cell lines (*n* = 18, PRJEB41558). Dataset was normalised to transcripts-per-million and a signature matrix was generated using CIBERSORTx.^25^

GTML genetically engineered mouse model (GEMM) and allograft profiles were applied to the signature to estimate cell-type fractions. Immune cell differences between diagnosis and recurrence were assessed using *t*-tests (ggpubr) and visualized with ggplot2.

### Microarray Data, Analysis, and Availability

Differential expression analysis was performed on 12 paired diagnostic and relapse MB tumors from the MAGIC consortium (GSE63670),^27^ profiled using Illumina 450k methylation arrays and normalized via SWAN (minfi v1.12.0).

Additionally, differential gene expression analysis of 763 diagnostic MB samples (subgrouped and subtyped, GSE85218) was conducted using Affymetrix Gene 1.1 ST arrays and 450 k methylation arrays. Data from Cavalli et al.^28^ is available at GEO: GSE85218.

### RNA-Seq Data Analysis (Ayrault Cohort)

RNA-sequencing analysis by MB subgroup/subtype was performed on 341 diagnostic samples from the Ayrault Lab, classified via the molecularneuropathology.org platform.^29^ Raw data were processed using an in-house pipeline from Institut Curie (https://github.com/bioinfo-pf-curie/RNA-seq), following standard protocols.

Counts were normalized using the variance stabilizing transformation (vst) from DESeq2. The dataset will be available in the EGA repository upon publication.

### Proteome Data Analysis (Ayrault Cohort)

Proteomic profiling of 343 diagnostic MB samples from the Ayrault Lab was analyzed by subgroup/subtype using the *molecularneuropathology.org* classifier. Mass spectrometry was performed in data-acquisition mode. Raw files were processed with Spectronaut 17 using an in-house spectral library, then normalized with myProMS v3.10^30^ (https://github.com/bioinfo-pf-curie/myproms). Data will be available in the PRIDE repository upon publication.

### CRISPR-Cas9-Mediated Knockout of MIF

**pFUGW.Cas9-Blast** (gift from the Schramek Lab) encodes spCas9 and a blasticidin resistance gene separated by a 2A peptide in a third-generation lentiviral backbone. Lentivirus was produced as previously described.^16^ Primary and recurrent GTML cells were transduced at MOI 5 and cultured for 7-days in Neurobasal medium with N21, N2 (Biotechne), 10 ng/ml EGF, 10 ng/ml bFGF (Stem Cell Technology), 2 μg/ml heparin (Sigma), and 10 μg/ml blasticidin.

**Guide RNAs** targeting MIF or a scrambled control were cloned into a third-generation pLKO stuffer lentiviral plasmid with mCherry (marker) and a U6-promoter. Lentivirus was produced and used to transduce GTML.Cas9 cells at MOI 5. After 7-days in vitro, cells were sorted for mCherry using FACSMelody (BD), expanded for 7 more days, and used for in vivo experiments as previously described. Guide RNA targets, sequences and specificity in Supplementary Methods.

### In Vitro Bone-Marrow Derived Macrophages

Bone marrow culture and polarization was adapted from Bailey et al.^31^ Bone marrow was harvested from 6 to 16-week-old mice by flushing femurs with PBS and filtering through a 70 μm strainer. Cells were pelleted (400 rcf, 5 min), resuspended in 90% FCS/10% DMSO, and frozen at –70 °C. On day-0, cells were thawed and plated at 2 × 10^4^/cm² in DMEM/F12 with 5% FCS and 25 ng/mL M-CSF. On day-5, non-adherent cells were removed, and fresh media with 50 ng/mL M-CSF were added. On day-6, 50 ng/mL GM-CSF was added. On day-7, cells were washed and polarized to M0: M-CSF only; M1: M0 + 100 ng/mL LPS + 25 ng/mL IFNγ; M2: M0 + 40 ng/mL IL-4. Were applicable, peptide (10 μg/mL) was added during polarization. After 24 h, cells were harvested with 5 mM EDTA at 37 °C for 30 min and analyzed by flow cytometry.

### Immunohistochemistry Processing and Staining

Tumors were fixed in 10% formalin for 24 h, transferred to 70% ethanol, and embedded as FFPE blocks. H&E slides were stained with hematoxylin (10 min), washed, and counterstained with 1% eosin (5 min). For IHC, goat anti-rabbit (Abcam) was used as the secondary antibody. Antibody and staining conditions are outlined in Supplementary Methods. All slides were developed with the DAB Map kit (8 min), counterstained (8 min), and scanned using the Hamamatsu NanoZoomer S360.

### Single-Cell RNA-Seq Data Analysis

Single cell RNA-sequencing (scRNA-seq) data were processed with CellRanger (v4.0.0) for alignment and UMI counting against the hg19 genome. Cells with fewer than 200 genes were excluded to remove empty droplets and low-quality cells. Filtered data were analyzed using Seurat (v4.3.0),^32^ with normalization via SCTransform.^33^ PCA was performed, followed by kNN graph construction. Libraries within each group were integrated by repeating normalization and principal component analysis (PCA). Cell cycle scores were calculated, and batch effects were corrected using Harmony (v0.1.1).^34^ The top 15 PCs were used for UMAP projection. Clustering was done with Seurat’s *FindNeighbours* and *FindClusters* (resolution = 1.5). Marker genes were identified using *FindAllMarkers* (logfc.threshold = 0.2, min.pct = 0.2), and clusters were manually annotated using known cell type markers.

### CellCrossTalker for Predicting Unbiased Cell-Cell Communications in Group3 and Group-4 MB

To study cell-cell communication in MB, we developed *CellCrossTalker*, a method using the discrete generalized beta distribution (DGBD) to model ligand and receptor expression across cell types. Expression ranks, rather than raw values, were used to improve noise tolerance and handle dropouts.^35^ UMI counts were normalized using Trimmed Mean of M-values (TMM) (edgeR), followed by Voom transformation.^36^ Expression values were scaled and binned; DGBD was then fitted to ranked bins using maximum likelihood estimation via the R optim function. The discrete generalized beta model was applied to these sorted expression ranks with frequencies λ, and two shape parameters α and β. The mass probability $p_{\lambda }$ of the DGBD for the λ*-th* rank that have $z_{\lambda }$ cell frequencies for a given ligand or receptor, see **Supplementary Methods**. This approach was applied across all ligand-receptor pairs to predict interactions, with a focus on tumor–immune cell communication.

## Results

### Transcriptional Signatures of Infiltrating Immune Cell Types in Relapsed Medulloblastomas Reflect Those Observed at Diagnosis

**Figure 1.**
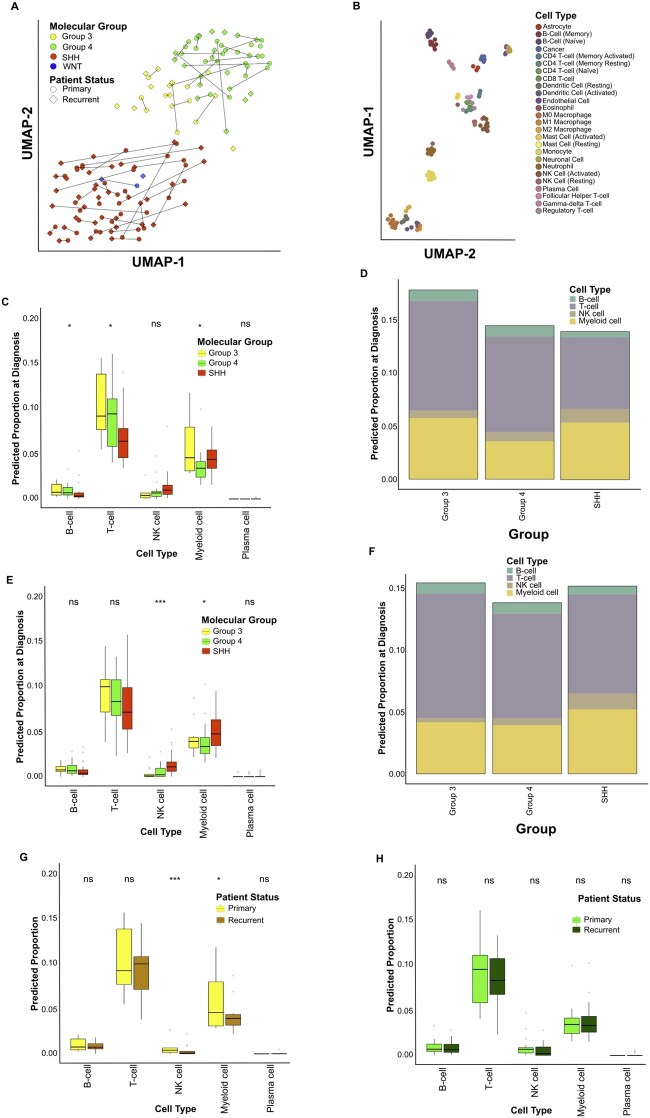
The predominant immune cell types present within diagnostic and relapse MB, and the interplay between the two disease stages. (A) UMAP plot of RNA-sequencing data for combined relapse cohort (*n* = 140, diagnostic-relapse pairs = 58). Diagnostic-Relapse pairs are joined by lines, colored by MB molecular group; WNT = Blue, SHH = Red, Group3 = Yellow, Group4 = Green. Point shape represents cohort; RNA-Sequencing (this study) = Triangle, RNA-Capture (this study) = Diamond, Okonechnikov = Circle. (B) UMAP plot depicting the deconvolution signature set used in this study. The signature set is composed of the LM22 dataset (*n* = 112) combined with cancer cell lines (*n* = 8), astrocytes (*n* = 3), endothelial (*n* = 4) and neuronal cells (*n* = 6). (C) Box and whisker plot of immune cell-group proportion estimates for diagnostic patient samples (*n* = 62) by MB diagnostic molecular group; SHH = Red, Group3 = Yellow, Group4 = Green. Statistical differences between groups were calculated using the one-way ANOVA test, where; ns = not significant, * = <0.05, ** = <0.005, *** = 0.0005. (D) Stacked bar chart showing mean immune cell-group proportion estimates for diagnostic patient samples (*n* = 62) by MB diagnostic molecular group. (E) Box and whisker plot of immune cell-group proportion estimates for relapse patient samples (*n* = 76) by MB diagnostic molecular group; SHH = Red, Group3 = Yellow, Group4 = Green. Statistical differences between groups were calculated using the one-way ANOVA test, where; ns = not significant, * = <0.05, ** = <0.005, *** = 0.0005. (F) Stacked bar chart showing mean immune cell-group proportion estimates for diagnostic patient samples (*n* = 76) by MB diagnostic molecular group. (G) Box and whisker plot of immune cell-group proportion estimates for MB Group3 diagnostic and relapse tumor samples (*n* = 20, diagnostic = 6, relapse = 14). Statistical differences between groups were calculated using *t*-test, where; ns = not significant, * = <0.05, ** = <0.005, *** = 0.0005. (H) Box and whisker plot of immune cell-group proportion estimates for MB Group4 diagnostic and relapse tumor samples (*n* = 44, diagnostic = 23, relapse = 21). Statistical differences between groups were calculated using *t*-test, where; ns = not significant, * = <0.05, ** = <0.005, *** = 0.0005.

Intra-tumoral heterogeneity within the primary and recurrent TME may contribute to disease maintenance and progression. To explore this hypothesis, we utilized a comprehensive RNA-sequencing dataset (Tables S1A-C and S2) comprising paired diagnostic and relapsed cases stratified by molecular subgroup (*n* = 54 in-house samples, *n* = 86 external samples, Table S2).^10^ As anticipated,^11^^,^^13^ MB principal molecular subgroups were maintained at recurrence, emphasizing their stability (Figure 1A). To assess the relative inferred proportion of immune cells between the primary and recurrent Group3 and Group4-MBs, we combined the LM22 immune cell dataset^23^ with MB cell lines (*n* = 8), astrocytes (*n* = 3), brain endothelial cells (*n* = 4) and neuronal cells (*n* = 6) (Figure 1B); SHH MB served as a control group due to the well-documented immune infiltration.^6^

At diagnosis, Group4-MBs showed significantly higher T-cell infiltration compared to SHH-MB (Mean Group4 infiltrate = 9.1%, Mean SHH infiltrate = 6.7%, padj = 0.047); however, Group3-MB did not differ significantly (Mean Group3 infiltrate = 10%, padj = 0.119) (Figure 1C and D; Figure S1A-D). As expected,^37^ SHH-MB demonstrated higher myeloid infiltration than Group4 (Mean SHH infiltrate = 5.4%, Mean Group4 infiltrate = 3.6%, padj = 0.025), however, only monocytes differed significantly between Group3-MB and SHH-MB (Mean SHH infiltrate = 2.3%, Mean Group3 infiltrate = 0.3%, padj = 0.001) (Figure S2A-D).

At recurrence, T-cell levels were similar across all molecular groups (Mean SHH infiltrate = 8%, Mean Group3 infiltrate = 10%, Mean Group4 infiltrate = 8.4%) (Figure 1E and F; Figures S1E and F). However, SHH-MB retained higher myeloid levels than Group4-MB (Mean SHH infiltrate = 5.2%, Mean Group4 infiltrate = 3.4%, padj = 0.044), with only monocytes differing between Group3-MB and SHH-MB (Mean SHH infiltrate = 1.9%, Mean Group3 infiltrate = 0.8%, padj = 0.012; Figure S2E and F), indicating sustained myeloid cell infiltration in both subgroups throughout tumor progression.

Within-subgroup showed no significant changes in immune cell-class infiltrate (Figures 1G and H; Figures S2G and H). However, regulatory T-cell infiltration decreased in Group4-MB at recurrence (diagnosis = 0.9%, recurrence = 0.5%, padj = 0.050), while memory resting CD4 T-cells increased in Group3-MB recurrence (primary = 0.5%, recurrence = 2%, padj = 0.010) (**Figures 1G and H**). All other cell types remained stable (non-significant), suggesting immune cell maintenance during progression at the transcriptomic level.

### Relapsed Medulloblastomas Exhibit Spatial Interactions Characteristic of an Immune-Suppressed Microenvironment

To investigate whether Group3 and Group4-MB share immune cell infiltration at diagnosis and relapse, we conducted spatial immunophenotyping^38^ on paired cases. This analysis aimed to elucidate spatial relationships among immune cell phenotypes, highlighting potential interaction or segregation patterns. Our spatial interactions analysis included eight relapse pairs from two independent cohorts, enhancing analytical power and enabling cross-validation (Table S2). Deconvolution analysis of a larger integrated dataset, combining our in-house (*n* = 54) and external MB samples (*n* = 86),^10^ provide a high-resolution insight into immune composition. Together, these complementary datasets offer a comprehensive view of immune infiltration in MB, particularly at relapse.

**Figure 2.**
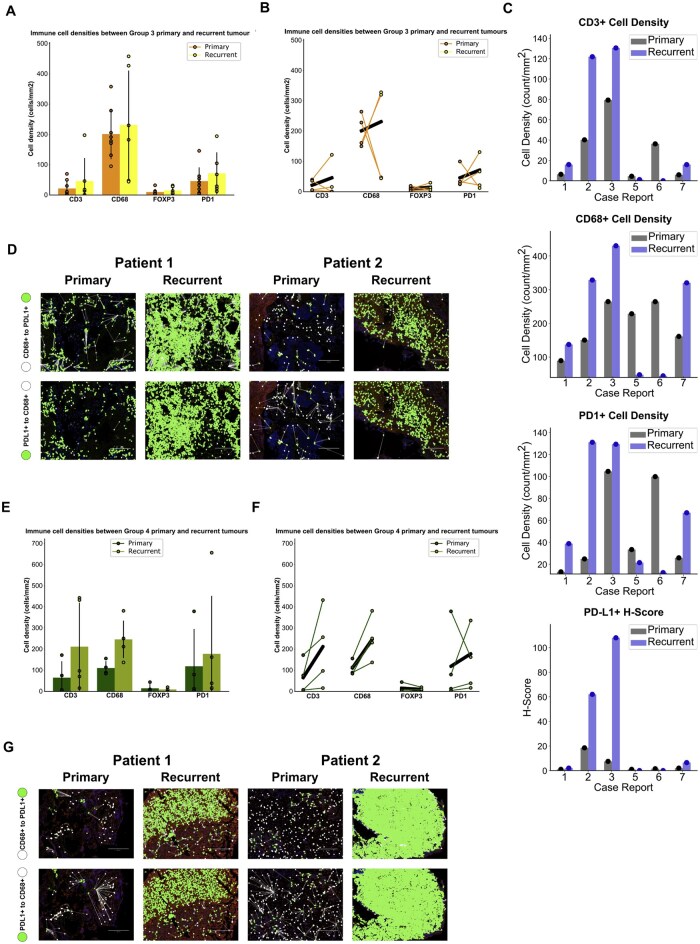
Phenotypic immune signatures and spatial profiles observed in diagnostic and relapse tumors of Group3 and Group4 MB. (A) Immune cell densities (cells/mm^2^) in four matched Group3 MBa diagnostic and relapse tumor cases. Mean case comparisons of diagnostic and relapse tumor samples show a trending increase in CD3, FoxP3 and PDL1 cell densities in the relapse tumor compartment, and maintenance of CD68 between diagnostic and relapse tumors. Statistical significance for a and b was calculated using one-way ANOVA with Tukey’s post-test, Shapiro-Wilk test used to determine normal distribution of data. Data represents 4 biological replicates. (B) Four matched pairs illustrating immune cell densities between diagnostic and relapse tumors in Group3 MB. Mean case comparisons of diagnostic and relapse tumor samples further demonstrate a trending increase in CD3, FoxP3, and PDL1 cell densities in the relapse tumor compartment, and maintenance of CD68 between diagnostic and relapse tumors. Connecting lines to denote diagnostic-relapse pairs. Statistical significance for a and b calculated using one-way ANOVA with Tukey’s post-test, Shapiro-Wilk test used to determine normal distribution of data. Data represents 4 biological replicates. (C) Direct case comparisons of diagnostic vs. relapse tumor samples showed three of the five markers having increased cell densities in the relapse MB samples: CD3, CD68, and PD-1. In addition, PD-L1 also showed an increased presence from diagnostic to relapse tumor samples. (D) Nearest neighbor spatial phenotypic cell analysis comparing diagnostic and relapse tumors in two independent cases of Group3 MB patients. Connecting lines denote different cell phenotypes (colored circles denote each phenotype) to better elucidate the spatial relationships between cell phenotypes, and phenotypic differences between tumor regions. Shown are one-way directional cell-to-cell interactions superimposed on a multiplex immunofluorescence field-of-view, with nearest CD68+ (white circle) to each PDL1+ cells (green circle), nearest PDL1+ (green circle) to each CD68+ (white circle) cells. Scale bar represents 200 µM; data represents 4 biological replicates. (E) Comparison of immune cell densities (cells/mm^2^) between four matched Group4 MB diagnostic and relapse tumor cases. Mean case comparisons of diagnostic and relapse tumor samples show a trending increase in CD3, CD68 and PDL1 cell densities in the relapse tumor compartment, and maintenance of FoxP3 between diagnostic and relapse tumors. Statistical significance for a and b calculated using one-way ANOVA with Tukey’s post-test, Shapiro-Wilk test used to determine normal distribution of data. Data represents 4 biological replicates. (F) Four matched pairs illustrating immune cell densities between diagnostic and relapse tumors in Group4 MB. Mean case comparisons of diagnostic and relapse tumor samples further demonstrate a trending increase in CD3, CD68 and PDL1 cell densities in the relapse tumor compartment, and maintenance of FoxP3 between diagnostic and relapse tumors. Connecting lines to denote diagnostic-relapse pairs. Statistical significance for a and b calculated using one-way ANOVA with Tukey’s post-test, Shapiro-Wilk test used to determine normal distribution of data. Data represents 4 biological replicates. (G) Spatial phenotypic analysis comparing diagnostic and relapse tumors in two independent cases of Group4 MB patients. Connecting lines denotes nearest neighbor analysis to elucidate the spatial relationships and phenotypic differences between tumor regions. Nearest neighbor analysis spatial analysis displays one-way directional cell-to-cell interactions, with nearest CD68+ (white circles) to each PDL1+ cells (green circles), nearest PDL1+ (green circles) to each CD68+ cells (white circles). Scale bar represents 200 µM; data represents 4 biological replicates.

In Group3-MBs, CD68+ tumor-associated macrophages (TAMs), exhibited the highest cellular density (cells/mm²), followed by programmed death receptor 1 (PD-1) positive cells (Figure 2A). consistent with our deconvolution results (Figure 1G, Figure S1H) demonstrating elevated myeloid cell infiltration. This pattern was evident in the diagnostic and matched relapsed tumor (Figure 2B and C). Moreover, relapsed Group3-MB displayed increased interactions between CD68+ cells and programmed death receptor ligand 1 (PDL1)+ cells, indicating a heightened spatial relationship (Figure 2D).

In Group4-MB, cellular densities (cells/mm^2^) for total T-cells (CD3), CD68, and PD1 showed an increase within the relapse tumors (Figure 2E) and consistently observed across all matched diagnostic and relapsed tumors (Figure 2F). Moreover, all four paired cases (two shown) exhibited a heightened spatial relationship between CD68+ and PDL1+ cells at relapse tumor compared to the diagnostic disease (Figure 2G). Our findings indicate that Group4 tumors exhibit significant changes in the relapsed TME compared to Group3 tumors, which maintain consistent cell densities. The spatial relationship of CD68^+^ and PD-L1^+^ within relapse Group3 and Group4-MBs indicates a more pronounced immunosuppressive TME,^39^ suggesting active immune evasion by PD-L1-mediated inhibition of T-cell activity while recruiting CD68^+^ macrophages polarized to an M2-like phenotype.^40^ These findings highlight the therapeutic potential of targeting CD68+ macrophages, to reduce immune suppression and enhance the immune system’s ability to fight the tumor.^41^^,^^42^

### The CD74-MIF Axis is a Prominent Tumor-Immune Interaction within Medulloblastoma

To investigate therapeutically targetable intercellular communications in the TME of Group3 and Group4-MB, we utilized 10X MB single-cell data and advanced novel algorithms (Figure S3A), to predict prominent ligand-receptor interactions between tumor cells and immune cells within the TME.

**Figure 3.**
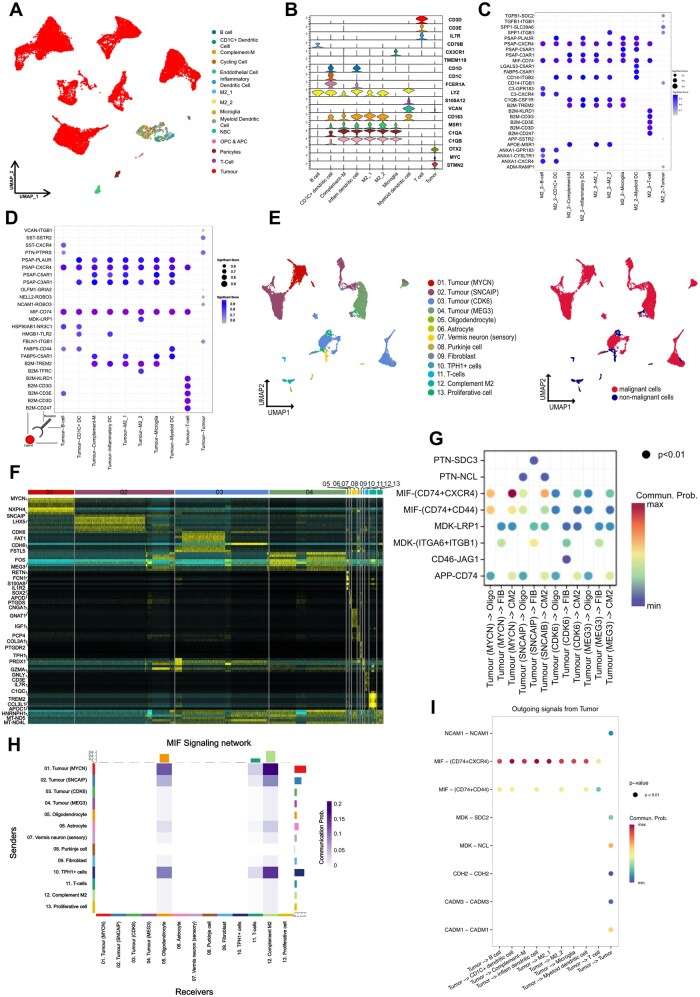
Cell-to-cell communications within the TIME for human Group3 and Group4 MB. (A) The UMAP plot of cell type clusters show the identification of 15 distinct cell clusters from the single-cell RNA-seq data of Group3 MB samples. OPC: Oligodendrocyte Precursor Cell. APC: Astrocyte Precursor Cell. M2_1: M2 macrophages cluster 1. M2_2: M2 macrophages cluster 2. NSC: Neural stem cell. Complement-M, complement macrophage. inflam dendritic cell: inflammatory dendritic cell. (B) The violin plot of the marker gene expression for each cell cluster. (C) CellCrossTalker predicted ligand-receptor-mediated tumor-immune cell communications using scRNA-seq data from Group3 MB samples. The vertical axis represents ligands and their corresponding receptors, while the horizontal axis indicates the sender cell type associated with the ligand and the receiver cell type associated with the receptor. (D) CellCrossTalker predicted ligand-receptor-mediated communications between M2 macrophages and tumor cells, M2 macrophages and T cells, M2 macrophages and B cells, as well as M2 macrophages and myeloid cells, using scRNA-seq data from Group3 MB samples. The vertical axis represents ligands and their corresponding receptors, while the horizontal axis shows the sender cell type associated with the ligand and the receiver cell type associated with the receptor. (E) The UMAP plot of cell type clusters show the identification of 13 distinct cell clusters. Single-cell RNA-seq data from Group4 MB samples were obtained from Hendrikse et al.^1^ (F) The heatmap illustrates the expression of marker genes used to annotate each cell cluster. (G) CellCrossTalker predicted ligand-receptor-mediated cell-cell communications using scRNA-seq data from Group4 MB samples. The vertical axis represents ligands and their corresponding receptors, while the horizontal axis shows the sender cell type associated with the ligand and the receiver cell type associated with the receptor. (H) The heatmap shows the interaction strength of MIF-CD74 between various pairs of cell types, as predicted by CellCrossTalker using scRNA-seq data from Group4 MB samples. (I) CellCrossTalker predicted ligand-receptor-mediated communication co-receptors between tumor cells and immune compartment, using scRNA-seq data from Group3 and Group4 MB samples. The vertical axis represents ligands and their corresponding receptors and co-receptors, while the horizontal axis shows the sender cell type associated with the ligand and the receiver cell type associated with the receptor.

Six Group3-MB samples^7^ (BT2016012, BT2018034, BT2018086, BT2019011, MBT058, and PS17-3376)^7^^,^^43^ with characterized MYC status (amplification/balanced; Supplementary 3B) were annotated using Seurat^32^ and visualized using the UMAP method, resulting in a total of 21,802 cells (Figure 3A) and 15 distinct cell type clusters (Figure 3B). To predict prominent cellular interactions within the TME, we developed the *CellCrossTalker* tool and applied it to these 15 cell clusters to predict unbiased ligand-receptor interactions between the tumor and immune cells, signaling pathways, and communication networks across various cell types within the TME.

Our analysis uncovered a prominent macrophage migration inhibitory factor (MIF)-CD74 interaction mediated by tumor cells with multiple immune and glial cell populations, including T-cells, B-cells, M2-macrophages, myeloid dendritic cells, inflammatory dendritic cells, and microglia (Figure 3C). Notably, M2-macrophages also mediated MIF-CD74 signaling with T-cells, B-cells, myeloid dendritic cells, inflammatory dendritic cells, and microglia (Figure 3D), underscoring the immunosuppressive complexity within the TME.

We extended our analysis to eight Group4-MB samples,^43^ revealing 13 distinct cell type clusters (Figure 3E-F). Our *CellCrossTalker* tool revealed significant MIF-CD74 interactions between tumor cells and complement-associated M2-macrophages (Figure 3G and H), suggesting these macrophages play a central role shaping the immune microenvironment of Group4-MB. Annotated cell types highlight the marker gene expression across different cell types.

These findings suggest a conserved MIF-CD74 signaling axis, mediating tumor-immune interactions across MB subgroups, with distinct cellular participants depending on the tumor context. Notably, CD74 is known to promote M2-like polarization^44^ of CD68^+^ macrophages, enhancing PD-L1 expression and fostering an immunosuppressive TME and tumor progression. Protein-protein interaction analyses identified CXCR4 and CD44 as the most likely co-receptors mediating the MIF-CD74 interaction in both Group3 and Group4-MB (Figure 3I; Figure S3C-F).^45^ Consistent with activation of downstream MIF-CD74 signaling, M1-macrophages isolated from MIF-expressing primary GTML tumors displayed elevated phosphoERK levels together with reduced phospho-NFκB. Given that sustained ERK activation coupled with suppressed NFκB signaling can reprogram macrophages toward an immunoregulatory phenotype,^46^ these findings suggest that MIF secretion by tumor cells may blunt macrophage effector function and promotes a tumor-supportive immune microenvironment (Figure S3G). Therapeutically targeting the MIF-CD74 interaction could enhance anti-tumor immunity of Group3 and Group4-MB.

### Validation of CD74 as a Candidate Target in Medulloblastoma

**Figure 4.**
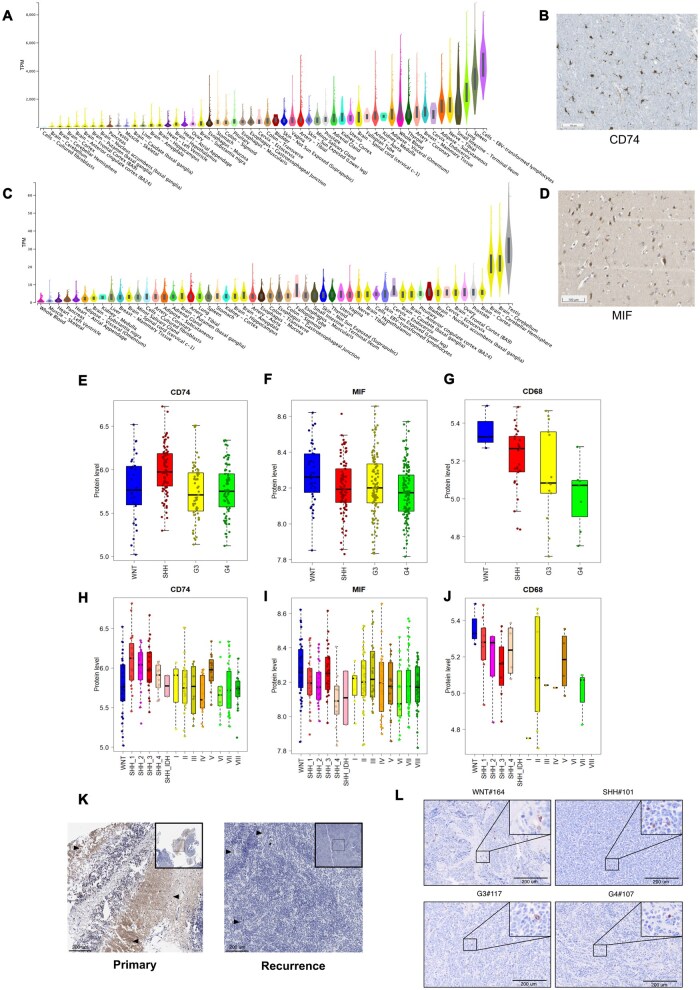
Restricted expression of CD74 and MIF in human normal tissues and MB subgroups. (A) Graph depicting the expression of *CD74* (ENSG00000019582.14) in normal human tissue RNA-sequencing data obtained from the GTEx consortium. The dataset comprises 7859 samples across 31 distinct normal tissues, with sample sizes ranging from 5 to 1152 samples per tissue. Expression levels are presented as relative expression levels in transcripts per million (TPM). (B) CD74 protein expression in normal human cerebellum. Scale bar, 100 um. (C) Graph depicting the expression of *MIF* (ENSG00000240972.1) in normal human tissue RNA-sequencing data obtained from the GTEx consortium. The dataset comprises 7859 samples across 31 distinct normal tissues, with sample sizes ranging from 5 to 1152 samples per tissue. Expression levels are presented as relative expression levels in transcripts per million (TPM). (D) MIF protein expression in normal human cerebellum. Scale bar, 100 um. (E-J) Protein levels across human MB subgroups and subtypes (based on DNA methylome classification) from Ayrault cohort for the three selected proteins: CD74, CD68 and MIF. Boxplots show median (line), upper and lower quartiles (boxes), and lines extending to highest and lowest observations (whiskers). (K) CD74 immunohistochemistry staining analysis of paired human pediatric diagnostic (left) and relapse (right) MB samples. Black arrows depict CD74 positivity. Scale bar represents 100 µM. (L) Immunohistochemistry membrane staining depicting CD74 expression across a subgrouped human diagnostic MB tissue microarray. Scale bar represents 200 µM.

To validate the MIF-CD74 pathway as a therapeutic target for Group3 and Group4-MB, analyzed CD74 and MIF RNA expression across normal healthy tissues from the GTEx consortium^47^ (*n* = 7859 samples across 31 unique normal tissues, with between 5 and 1152 samples per tissue) and validated the findings in normal brain tissue (*n* = 8 cerebral cortex; http://www.proteinatlas.org/). CD74 demonstrated minimal expression across all normal brain compartments (Figure 4A), with expression restricted to microglia and macrophages (Figure 4B), while MIF expression appeared highest across brain compartments (Figure 4C) (TPM > 40) MIF is known to be inducibly secreted by immune cells^48^ with limited protein localization detected in Purkinje cells and granular layer cells of normal pediatric cerebral cortex (Figure 4D), suggesting minimal off-target effects.

We observed protein and RNA level expression of CD74, MIF, and CD68 across all MB molecular subgroups and subtypes (Figure 4E-J; Figure S4A-F) with gene expression analysis supporting these findings (Figure S4G-I). Given the biological divergence of R-MB from its diagnostic tumor counterpart,^12^^,^^13^ we compared matched Group3 and Group-MBs at diagnostic and relapse, finding conserved expression of CD74, MIF and CD68 (Figure 4K; Figures S4J-L and S5A). CD74 protein expression was localized to TAMs and microglia in a tissue microarray^49^ of defined MB subgroups (Figure 4L; Figure S5B). The consistent and elevated expression of CD74 in both diagnostic and metastatic/relapse Group3 and Group4-MB, and contrasted low expression in normal developing tissues, highlights CD74 as a promising therapeutic target. Targeting CD74 may overcome immune suppression and restore anti-tumor immunity.

### A Model of Relapsed MYCN-Driven Medulloblastoma

**Figure 5.**
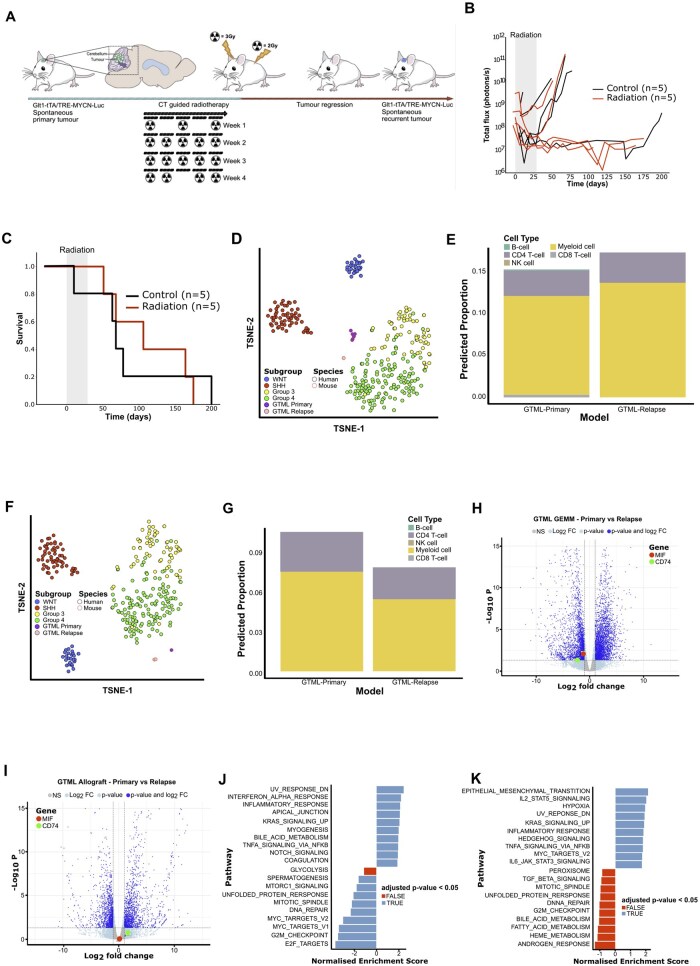
Prevailing immune cell types observed in a spontaneous primary MYCN MB murine model compared with its radiotherapy-treated counterpart. (A) Schematic representation of CT guided radiotherapy delivery in GTML GEMMs. Mice received radiation at doses of 3 Gy to the tumor bed and 2 Gy to the spine. The treatment regimen included 18 fractions, administered with a schedule of 5 consecutive days of therapy followed by 2 days off. Tumor relapse was monitored weekly using bioluminescence imaging (BLI). (B) Tumor burden over time of GTML GEMMS treated with radiotherapy (*n* = 5; red lines) or sham irradiation (*n* = 5; black lines) as expressed by total flux (photons per second) from bioluminescence imaging. Each line represents an individual mouse with the shaded area illustrating the treatment period; each dot (black or red) represents a bioluminescent imaging measurement. (C) Survival analysis of GTML GEMMs treated with radiotherapy (*n* = 5; red lines) or sham irradiation (*n* = 5; black lines). Data were analyzed by the Cox proportional hazard model. (D) TSNE projection of GTML GEMM mouse model RNA-Sequencing data (*n* = 9, diagnostic = 7, relapse = 2) onto diagnostic human MB cohort (*n* = 594). Projection was created using vst normalized expression data, subset to orthologous protein encoding human-murine genes (*n* = 16 242). Human MB samples are colored by diagnostic molecular group; WNT = blue, SHH = red, Group3 = yellow, Group = green. GTML GEMM models of MB primary and Recurrence are colored according to status; Primary = purple, Recurrence = pink. (E) Stacked bar chart showing mean immune cell-group proportion estimates for GTML GEMM models of diagnosis (*n* = 5) and relapse (*n* = 5). (F) TSNE projection of GTML Allograft mouse model RNA-Sequencing data (*n* = 6) onto diagnostic human MB cohort (*n* = 594). Projection was created using vst normalized expression data, subset to orthologous protein encoding human-murine genes (*n* = 16 242). Human MB samples are colored by primary molecular group; WNT = blue, SHH = red, Group3 = yellow, Group = green. GTML Allograft models of MB Diagnosis and Recurrence are colored according to status; Primary = purple, Recurrence = pink. (G) Stacked bar chart showing mean immune cell-group proportion estimates for GTML allograft models of diagnosis (*n* = 3) and radiation resistance (*n* = 3). (H) Volcano plot of differentially expressed genes (*n* = 5435) identified between Primary and Relapse (primary versus relapse) GEMM GTML mouse models. Blue dots on the left denote significant differentially expressed genes with ≤1 log fold change and those on the right with ≥1 log fold change. (I) Volcano plot of differentially expressed genes (*n* = 2764) identified between Primary and Relapse (primary versus relapse) allograft GTML mouse models. Blue dots on the left denote significant differentially expressed genes with ≤1 log fold change and those on the right with ≥1 log fold change. (J) Gene set enrichment analysis (GSEA) of the differentially expressed genes identified between Primary and Relapse (primary versus relapse) GEMM GTML mouse models. Blue bars denote hallmark pathways that were significantly enriched following Benjamini-Hochberg correction (*P* < .05) and red that were not significant (*P* > .05). NES: Normalized enrichment score. (K) Gene set enrichment analysis of the differentially expressed genes identified between Primary and Relapse (primary versus relapse) allograft GTML mouse models using GSEA. Gene sets are organized into the top 10 and bottom 10 based on normalized enrichment score. Blue bars denote hallmark pathways that were significantly enriched following Benjamini-Hochberg correction (*P* < .05) and red that were not significant (*P* > .05). NES: Normalized enrichment score.

Previous studies demonstrate that CD74 expression on immune cells plays a critical role in regulating cancer cell activity.^50^ Notably, CD74 levels increase in response to cytokine stimulation across various cancers and following immune checkpoint inhibitor treatments,^50^ suggesting CD74 contributes to immune activation. Based on these findings, we hypothesized that CD74 inhibition could remodel the immunosuppressive TME and enhance immune-mediated tumor control in primary and R-MB. To test this, we developed a R-MB MYCN-driven mouse model (GTML; *Glt1-tTA/TRE-MYCN-Luc*) (Figure 5A-C), originally described by Hill et al.,^51^ that accurately mimics the immunosuppressed TME of R-MB. As expected,^11^^,^^13^ primary and R-MB retained gene expression profiles characteristic of the human disease, with subgroup stability maintained at relapse (Figure 5D).

Next, to evaluate the TME alignment between our experimental models and clinical samples, we investigated the immune infiltration profiles in GTML primary and R-MB. Consistent with our human data, myeloid cells and CD4+ T-cells were the predominant infiltrating immune cell types (Figure 5E; Figure S5C-D). Specifically, macrophages showed persistent infiltration across both tumor compartments, while Tregs exhibited a notable increase in the relapsed disease, possibly due to therapy-induced effects.^52^

To establish a robust platform for preclinical trials within an immune competent microenvironment, we developed syngeneic allograft models of our primary and relapse GEMMs (Figures S5E-4G). The syngeneic models retained gene expression profiles characteristic of the human disease^51^ and the original primary and relapse GEMMs (Figure 5F). Crucially, the relative immune proportions observed in the human disease were maintained (Figure 5G; Figure S5H-I). The relapse GEMM and syngeneic models exhibited numerous differentially expressed genes compared to the respective primary counterpart (Figure 5H-I), with significant enrichment of the PI3K/AKT signaling, chromatin modification, and cell cycle/DNA damage response pathways at the point of disease relapse (Figure 5J-K). These findings, which are also observed in the human disease,^11^^,^^13^ suggest that these pathways play a critical role in tumor progression and R-MB. Furthermore, the relapse GEMM displayed a pronounced inflammatory response following radiotherapy, mirroring the inflammatory processes observed in human disease.^53^ This response was also maintained in the syngeneic model, underscoring the relevance and accuracy of our models in replicating the human disease environment and response to treatment.

The R-MB mouse model exhibited distinct intra-tumoral heterogeneity^12^ (Figures S5E-4K), which highlights the model’s utility in studying tumor behavior and progression. This model will be instrumental to identify and evaluate potential therapeutic targets and understanding the mechanisms underlying treatment resistance and tumor progression, in a syngeneic immune-competent background.

### CD74 Inhibition Provides Anti-Tumor Immunity

**Figure 6.**
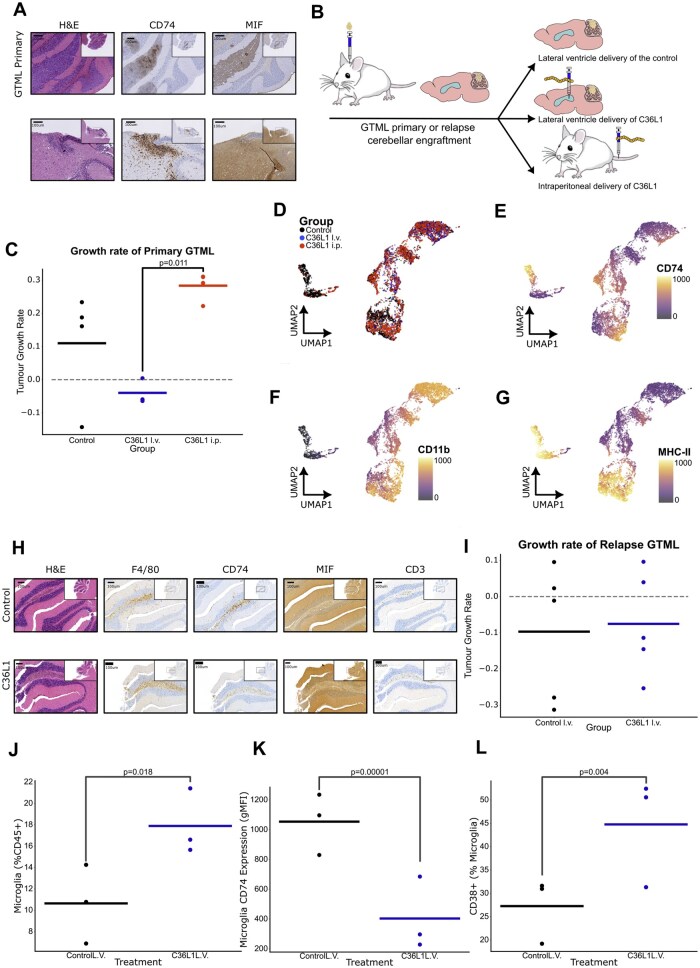
Inhibition of CD74 via the lateral ventricle demonstrates immune-modulation and decreased tumor burden in primary and relapsed MB. (A) IHC of treatment-naïve primary (top row) and radiation-treated (bottom row) GTML allografts displaying H&E, CD74, and MIF expression within the tumor. Results representative of 3 independent replicates. Much of the tumor outside the cerebellum was removed for downstream RNA sequencing analyses, Scale bar, 100 µm. (B) Experimental schematic for evaluating the CD74-MIF blocking peptide C36L1. GTML primary or relapse cells expressing firefly luciferase were allografted into the cerebellum of FVBNRJ mice. C36L1 was administered either intraperitoneally or locoregionally through the lateral ventricle at time of engraftment and 1-week after, C36L1 vehicle was used as a control. Bioluminescence imaging was conducted to monitor tumor engraftment, progression, and/or regression up to 14 days post-therapy. At the endpoint, the tumor and CNS were harvested and assessed for immune infiltration and tumor burden. (C) Growth rate of mice bearing primary GTML allografts (*n* = 3-4 per group) treated with vehicle control (black), C36L1 peptide through the lateral ventricle (red), or C36L1 peptide intraperitoneally (blue) was determined by calculating the slope of tumor growth between day 7 and 14 (endpoint). Line represents mean growth rate with individual points representing individual mice. Points below the dashed line indicate tumor regression. Statistical significance was calculated by two-way ANOVA with Tukey’s post-test. (D) UMAP projection of flow cytometry analysis of tumor/cerebellum of mice treated vehicle control or the C36L1 peptide via the lateral ventricle. The left panel display cells colored by experimental group (Control - black, C36L1 delivered via lateral ventricle - red, C36L1 delivered intraperitoneally - blue), highlighting the distribution and intensity target expression across different cell populations within the tumor and cerebellum of treated and control mice. The visualization provides insights into the immune cell infiltration and its association with the treatment groups. (E) UMAP projection of flow cytometry results, colored by the expression of CD74. (F) UMAP projection of flow cytometry results, colored by the expression of MHCII. (G) UMAP projection of flow cytometry results, colored by the expression of CD11b. (H) IHC of control (top row) and peptide-treated (bottom row) GTML primary tumor allografts displaying H&E, CD3, F4/80 and CD74 within the tumor. Results representative of 3 independent replicates, Scale bar, 100 um. (I) Growth rate of mice bearing recurrent GTML allografts (*n* = 5 per group) treated with vehicle control (black) or C36L1 peptide through the lateral ventricle (blue) was calculated by calculating the slope of tumor growth between day 7 and 14 (endpoint). Line represents mean growth rate with individual points representing individual mice. Points below the dashed line indicate tumor regression. Statistical significance was calculated by two-way ANOVA with Tukey’s post-test. (J) Bar chart to illustrate the proportion of tumor-associated immune cells identified as microglia. A higher proportion of microglia is observed in the TME of CD36L1 peptide treatment versus scrambled control. (K) Bar chart to illustrate the level of CD74 expression in the TME of mice treated with CD36L1 versus scrambled control. (L) Bar charts displaying the proportion of CD38+ cells in the microglia population. Statistical analysis was performed using a two-way ANOVA with Tukey’s post-test to compare the groups. Error bars represent the SD. Significant differences between groups are indicated by *P < .05, **P < .01, and ***P < .001.

Disrupting the MIF-CD74 signaling pathway in macrophages has been shown to restore anti-tumor immune responses by overcoming immune suppression in the TME.^50^ Building on these findings, we hypothesized that targeting CD74 could potentially restore anti-tumor immunity in MB. As MIF is expressed on tumor cells, CRISPR-Cas9 MIF knockout studies revealed a short-term reduction in tumor growth (Figure S6A and B). In primary GTML MIF K/O tumors, increased CD4^+^ memory T-cell populations together with reduced B-cell abundance (*P* < 0.005) indicate a shift toward a pro-inflammatory, anti-tumorigenic microenvironment. The expansion of CD4^+^ memory subsets (*P* < 0.05) likely enhance antigen-specific immune responses, while depletion of immunosuppressive B-cell populations (*P* < 0.005) may relieve T-cell inhibition and further promote immune activation (Figure S6C and D). In relapse GTML, MIF K/O tumors reduced naïve T-cell frequencies alongside expansion of effector and memory T-cell subsets similarly reflect heightened immune activation and a pro-inflammatory, anti-tumorigenic milieu (Figure S6C and D). Further to our knockout experiments, an Ig-CDR-derived peptide, C36L1, has shown efficacy in binding to CD74, thereby inhibiting MIF signaling and reducing the expression of immunosuppressive factors in syngeneic models of metastatic melanoma.^50^ Our research and that of others have previously illustrated significant therapeutic responses with locoregionally delivered chimeric antigen receptor (CAR) T-cells.^14^ Therefore, we sought to determine whether intraventricularly delivered C36L1 via the lateral ventricle (LV) could restore a level of immunity within our syngeneic mouse models of primary and R-MB (Figure 6A and B).

Surprisingly, in primary tumors, LV delivered C36L1 led to a significant reduction in tumor burden (*P* < 0.005) without any treatment-associated toxicities (Figure 6C; Figure S7A). Unsupervised clustering revealed enrichment of CD74^low^/MHC-II^low^ cells in treated mice (Figure 6D-F; Figure S7B and C). CD74 inhibition decreased M2-like macrophage polarization, promoting a pro-inflammatory macrophage phenotype characterized by CD11b^+^/F4/80^+^/CD206^-^ markers (Figure 6G; Figure S7D and E). This shift was accompanied by an increase in CD3 expression (Figure S7F), suggesting a transition toward a pro-inflammatory signature. Immunohistochemistry analysis of resulting tumors (where feasible due to tumor regression) confirmed these findings (Figure 6H), suggesting that CD74 inhibition may enhance pro-inflammatory pathways, aiding in tumor regression. *In vitro* experiments revealed that C36L1 treatment reprograms primary macrophages from an anti-inflammatory to a pro-inflammatory phenotype, highlighting its potential to modulate immune responses within the TME (Figures S7G-I).

Next, we sought to assess the peptide’s efficacy in our R-MB model. Only tumors that demonstrated complete response following LV C36L1 treatment exhibited a marked increase in CD38^+^/CD11b^+^ macrophages and myeloid cells (Figure 6I-L; Figures S7G-K). Notably, these cells displayed a shift toward an M1-like pro-inflammatory response, indicating that inhibiting the MIF-CD74 axis may play a crucial role in reprogramming the tumor-associated immune response. Although no significant differences in longitudinal survival were observed between treated groups (Figure S8A-D), this supports our hypothesis that CD74-MIF blockade primarily modulates the TIME. These findings highlight the pathway’s potential to reshape immune composition, fostering an anti-tumor profile, and potentially enhancing the efficacy of subsequent immunotherapy strategies.

## Discussion

Patients with R-MB face dismal survival rates, regardless of the treatment modalities received.^11^ Previous studies have identified pivotal relapse-associated molecular events and divergent clonal selection at recurrence.^10^^,^^12^^,^^13^ Nonetheless, a limited understanding of the TIME and its role in disease progression has significantly impeded the development of effective therapeutic strategies. Here, we present the first comprehensive study to evaluate the TIME of diagnostic and R-MB.

Comprehensive analysis of immune cell proportions revealed few significant differences in the TIME between SHH, Group3, and Group4 subgroup tumors sampled at both diagnosis and recurrence. Notably, T-cell levels remained consistent across the molecular subgroups, while a sustained infiltration of myeloid cells was observed in SHH and Group3-MBs throughout tumor progression. These findings suggest that the immune cell composition is largely preserved during tumor progression, when assessed at the RNA level.

Spatial phenotypic analyses revealed a shift toward a highly immunosuppressive profile in relapsed Group3 and Group4-MB. Our findings indicate that Group4-MB exhibit more significant changes in the TME compared to Group3-MB, which maintain consistent cell densities. The spatial relationship between CD68^+^ macrophages and PD-L1^+^-expressing cells in relapsed Group3 and Group4-MB suggests a more pronounced immunosuppressive TME.^39^ This indicates that these tumors may actively evade immune surveillance by leveraging PD-L1-mediated inhibition of T-cell activity while recruiting CD68^+^ macrophages polarized to an M2-like phenotype, which are often associated with immunosuppressive functions.^54^ In animal models of breast cancer and glioma,^54^ macrophages have been shown to influence cancer cell behavior, facilitating invasion and metastasis. The observed enrichment of these phenotypes at relapse implies that immune evasion mechanisms may play a critical role in tumor persistence and progression, potentially contributing to therapeutic resistance and disease recurrence.

To ensure effective anticancer therapies, modifications to the TIME may be necessary to restore immunogenic functions. We developed a novel algorithm, *CellCrossTalker,* to identify critical ligand-receptor interactions between MB tumor cells and the immune microenvironment, enabling detailed analyses of the communication pathways that mediate tumor-immune interactions, tumor progression and immune evasion. Our analysis uncovered a prominent interaction between MIF and CD74, mediated by tumor cells within the TME. CD74 is a non-polymorphic type II transmembrane glycoprotein that acts as a high-affinity receptor for MIF.^55^ The MIF-CD74 axis has been identified as a potential therapeutic target in various cancers, including glioblastoma,^56^ and melanoma,^57^ where it is known to drive the functional polarization of macrophages toward an M2-like phenotype. Inhibition of the MIF-CD74 interaction can reverse this immunosuppressive environment, converting myeloid-derived suppressor cells to an immunostimulatory phenotype.^58^ Several modulators of the MIF-CD74 pathway are currently undergoing clinical trials^59^ for patients with metastatic colorectal adenocarcinoma, non-small-cell lung cancer, and ovarian cancer.

Using a newly characterized immune-competent MYCN-driven model of R-MB and its primary counterpart,^51^ we show that CD74 inhibition reduces M2-like macrophage phenotype and increases CD3^+^ T-cell infiltration, promoting a pro-inflammatory TIME. These results suggest that CD74 blockade can reprogramme the TME to support immune activation.

Our findings show diagnostic and R-MB are highly immune-compromised and confirm our algorithm’s ability to identify key tumor-immune interactions with therapeutic vulnerabilities. Targeting the MIF-CD74 axis as a priming approach, combined with other immunotherapies, could enhance anti-tumor immunity and warrants further translational development.

These strategies offer strong therapeutic potential for Group3 and Group4-MB throughout the disease course, addressing immune suppression and enabling more effective treatments. The MIF-CD74 interaction exemplifies broader targeting opportunities within these mechanisms and validates the effectiveness of our pipeline for wider therapeutic application.

## Supplementary Material

noag020_Supplementary_Data

## Data Availability

*Deconvolution analyses*: No custom code was used in this study. Open-source algorithms were used as detailed in the methods section. Details on how these algorithms were used are available from the corresponding authors upon request. *CellCrossTalker* tool: Original codes used for the study will be available at: Roosevelt-PKU/Tumor-immune-stromal-interaction. This study did not generate new unique reagents.
