# Supplementary material for: MIF-CD74 signaling drives immune modulation in medulloblastoma
Source: Neuro Oncol. 2026 Feb 6;28(5):1316–34. doi: 10.1093/neuonc/noag020 (PMC13186457; doi:10.1093/neuonc/noag020)
Supplement: noag020_Supplementary_Data [file noag020_supplementary_data.zip › Supplementary table 1.docx]

|  |  |  |  |  |
| --- | --- | --- | --- | --- |
|  | **Cell Type** | **Group** | **Mean** |  |
|  | ***B cells naïve*** | **SHH All (n=70)** | 0.0041 |  |
|  |  | **SHH Primary (n=33)** | 0.0030 |  |
|  |  | **SHH Recurrence (n=37)** | 0.0051 |  |
|  | ***B cells memory*** | **SHH All (n=70)** | 0.0022 |  |
|  |  | **SHH Primary (n=33)** | 0.0033 |  |
|  |  | **SHH Recurrence (n=37)** | 0.0013 |  |
|  | ***Plasma cells*** | **SHH All (n=70)** | 0.0006 |  |
|  |  | **SHH Primary (n=33)** | 0.0003 |  |
|  |  | **SHH Recurrence (n=37)** | 0.0008 |  |
|  | ***T cells CD8*** | **SHH All (n=70)** | 0.0296 |  |
|  |  | **SHH Primary (n=33)** | 0.0243 |  |
|  |  | **SHH Recurrence (n=37)** | 0.0344 |  |
|  | ***T cells CD4 naive*** | **SHH All (n=70)** | 0.0000 |  |
|  |  | **SHH Primary (n=33)** | 0.0000 |  |
|  |  | **SHH Recurrence (n=37)** | 0.0001 |  |
|  | ***T cells CD4 memory resting*** | **SHH All (n=70)** | 0.0270 |  |
|  |  | **SHH Primary (n=33)** | 0.0266 |  |
|  |  | **SHH Recurrence (n=37)** | 0.0273 |  |
|  | ***T cells CD4 memory activated*** | **SHH All (n=70)** | 0.0007 |  |
|  |  | **SHH Primary (n=33)** | 0.0005 |  |
|  |  | **SHH Recurrence (n=37)** | 0.0009 |  |
|  | ***T cells follicular helper*** | **SHH All (n=70)** | 0.0085 |  |
|  |  | **SHH Primary (n=33)** | 0.0075 |  |
|  |  | **SHH Recurrence (n=37)** | 0.0094 |  |
|  | ***T cells gamma delta*** | **SHH All (n=70)** | 0.0010 |  |
|  |  | **SHH Primary (n=33)** | 0.0004 |  |
|  |  | **SHH Recurrence (n=37)** | 0.0016 |  |
|  | ***T cells regulatory (Tregs)*** | **SHH All (n=70)** | 0.0069 |  |
|  |  | **SHH Primary (n=33)** | 0.0072 |  |
|  |  | **SHH Recurrence (n=37)** | 0.0065 |  |
|  | ***NK cells resting*** | **SHH All (n=70)** | 0.0077 |  |
|  |  | **SHH Primary (n=33)** | 0.0070 |  |
|  |  | **SHH Recurrence (n=37)** | 0.0083 |  |
|  | ***NK cells activated*** | **SHH All (n=70)** | 0.0052 |  |
|  |  | **SHH Primary (n=33)** | 0.0060 |  |
|  |  | **SHH Recurrence (n=37)** | 0.0046 |  |
|  | ***Monocytes*** | **SHH All (n=70)** | 0.0208 |  |
|  |  | **SHH Primary (n=33)** | 0.0229 |  |
|  |  | **SHH Recurrence (n=37)** | 0.0189 |  |
|  | ***Dendritic cells resting*** | **SHH All (n=70)** | 0.0014 |  |
|  |  | **SHH Primary (n=33)** | 0.0011 |  |
|  |  | **SHH Recurrence (n=37)** | 0.0018 |  |
|  | ***Dendritic cells activated*** | **SHH All (n=70)** | 0.0000 |  |
|  |  | **SHH Primary (n=33)** | 0.0000 |  |
|  |  | **SHH Recurrence (n=37)** | 0.0000 |  |
|  | ***Mast cells resting*** | **SHH All (n=70)** | 0.0035 |  |
|  |  | **SHH Primary (n=33)** | 0.0033 |  |
|  |  | **SHH Recurrence (n=37)** | 0.0037 |  |
|  | ***Mast cells activated*** | **SHH All (n=70)** | 0.0021 |  |
|  |  | **SHH Primary (n=33)** | 0.0024 |  |
|  |  | **SHH Recurrence (n=37)** | 0.0019 |  |
|  | ***Eosinophils*** | **SHH All (n=70)** | 0.0020 |  |
|  |  | **SHH Primary (n=33)** | 0.0027 |  |
|  |  | **SHH Recurrence (n=37)** | 0.0015 |  |
|  | ***Neutrophils*** | **SHH All (n=70)** | 0.0012 |  |
|  |  | **SHH Primary (n=33)** | 0.0023 |  |
|  |  | **SHH Recurrence (n=37)** | 0.0003 |  |
|  | ***Macrophage M0*** | **SHH All (n=70)** | 0.0042 |  |
|  |  | **SHH Primary (n=33)** | 0.0034 |  |
|  |  | **SHH Recurrence (n=37)** | 0.0050 |  |
|  | ***Macrophage M1*** | **SHH All (n=70)** | 0.0065 |  |
|  |  | **SHH Primary (n=33)** | 0.0061 |  |
|  |  | **SHH Recurrence (n=37)** | 0.0069 |  |
|  | ***Macrophage M2*** | **SHH All (n=70)** | 0.0106 |  |
|  |  | **SHH Primary (n=33)** | 0.0092 |  |
|  |  | **SHH Recurrence (n=37)** | 0.0118 |  |

|  |  |  |  |  |
| --- | --- | --- | --- | --- |
|  | **Cell Type** | **Group** | **Mean** |  |
|  | ***B cells naïve*** | **Grp3 All (n=20)** | 0.0088 |  |
|  |  | **Grp3 Primary (n=6)** | 0.0098 |  |
|  |  | **Grp3 Recurrence (n=14)** | 0.0084 |  |
|  | ***B cells memory*** | **Grp3 All (n=20)** | 0.0004 |  |
|  |  | **Grp3 Primary (n=6)** | 0.0004 |  |
|  |  | **Grp3 Recurrence (n=14)** | 0.0003 |  |
|  | ***Plasma cells*** | **Grp3 All (n=20)** | 0.0003 |  |
|  |  | **Grp3 Primary (n=6)** | 0.0001 |  |
|  |  | **Grp3 Recurrence (n=14)** | 0.0004 |  |
|  | ***T cells CD8*** | **Grp3 All (n=20)** | 0.0566 |  |
|  |  | **Grp3 Primary (n=6)** | 0.0775 |  |
|  |  | **Grp3 Recurrence (n=14)** | 0.0477 |  |
|  | ***T cells CD4 naive*** | **Grp3 All (n=20)** | 0.0000 |  |
|  |  | **Grp3 Primary (n=6)** | 0.0000 |  |
|  |  | **Grp3 Recurrence (n=14)** | 0.0000 |  |
|  | ***T cells CD4 memory resting*** | **Grp3 All (n=20)** | 0.0214 |  |
|  |  | **Grp3 Primary (n=6)** | 0.0055 |  |
|  |  | **Grp3 Recurrence (n=14)** | 0.0282 |  |
|  | ***T cells CD4 memory activated*** | **Grp3 All (n=20)** | 0.0023 |  |
|  |  | **Grp3 Primary (n=6)** | 0.0025 |  |
|  |  | **Grp3 Recurrence (n=14)** | 0.0023 |  |
|  | ***T cells follicular helper*** | **Grp3 All (n=20)** | 0.0156 |  |
|  |  | **Grp3 Primary (n=6)** | 0.0118 |  |
|  |  | **Grp3 Recurrence (n=14)** | 0.0172 |  |
|  | ***T cells gamma delta*** | **Grp3 All (n=20)** | 0.0006 |  |
|  |  | **Grp3 Primary (n=6)** | 0.0000 |  |
|  |  | **Grp3 Recurrence (n=14)** | 0.0009 |  |
|  | ***T cells regulatory (Tregs)*** | **Grp3 All (n=20)** | 0.0044 |  |
|  |  | **Grp3 Primary (n=6)** | 0.0054 |  |
|  |  | **Grp3 Recurrence (n=14)** | 0.0039 |  |
|  | ***NK cells resting*** | **Grp3 All (n=20)** | 0.0036 |  |
|  |  | **Grp3 Primary (n=6)** | 0.0057 |  |
|  |  | **Grp3 Recurrence (n=14)** | 0.0027 |  |
|  | ***NK cells activated*** | **Grp3 All (n=20)** | 0.0009 |  |
|  |  | **Grp3 Primary (n=6)** | 0.0013 |  |
|  |  | **Grp3 Recurrence (n=14)** | 0.0008 |  |
|  | ***Monocytes*** | **Grp3 All (n=20)** | 0.0068 |  |
|  |  | **Grp3 Primary (n=6)** | 0.0039 |  |
|  |  | **Grp3 Recurrence (n=14)** | 0.0081 |  |
|  | ***Dendritic cells resting*** | **Grp3 All (n=20)** | 0.0008 |  |
|  |  | **Grp3 Primary (n=6)** | 0.0024 |  |
|  |  | **Grp3 Recurrence (n=14)** | 0.0002 |  |
|  | ***Dendritic cells activated*** | **Grp3 All (n=20)** | 0.0001 |  |
|  |  | **Grp3 Primary (n=6)** | 0.0002 |  |
|  |  | **Grp3 Recurrence (n=14)** | 0.0000 |  |
|  | ***Mast cells resting*** | **Grp3 All (n=20)** | 0.0016 |  |
|  |  | **Grp3 Primary (n=6)** | 0.0018 |  |
|  |  | **Grp3 Recurrence (n=14)** | 0.0015 |  |
|  | ***Mast cells activated*** | **Grp3 All (n=20)** | 0.0068 |  |
|  |  | **Grp3 Primary (n=6)** | 0.0070 |  |
|  |  | **Grp3 Recurrence (n=14)** | 0.0067 |  |
|  | ***Eosinophils*** | **Grp3 All (n=20)** | 0.0014 |  |
|  |  | **Grp3 Primary (n=6)** | 0.0024 |  |
|  |  | **Grp3 Recurrence (n=14)** | 0.0010 |  |
|  | ***Neutrophils*** | **Grp3 All (n=20)** | 0.0018 |  |
|  |  | **Grp3 Primary (n=6)** | 0.0045 |  |
|  |  | **Grp3 Recurrence (n=14)** | 0.0006 |  |
|  | ***Macrophage M0*** | **Grp3 All (n=20)** | 0.0110 |  |
|  |  | **Grp3 Primary (n=6)** | 0.0179 |  |
|  |  | **Grp3 Recurrence (n=14)** | 0.0080 |  |
|  | ***Macrophage M1*** | **Grp3 All (n=20)** | 0.0066 |  |
|  |  | **Grp3 Primary (n=6)** | 0.0088 |  |
|  |  | **Grp3 Recurrence (n=14)** | 0.0056 |  |
|  | ***Macrophage M2*** | **Grp3 All (n=20)** | 0.0097 |  |
|  |  | **Grp3 Primary (n=6)** | 0.0100 |  |
|  |  | **Grp3 Recurrence (n=14)** | 0.0096 |  |
|  |  |  |  |  |

| **Cell Type** | **Group** | **Mean** |
| --- | --- | --- |
| ***B cells naïve*** | **Grp4 All (n=44)** | 0.0084 |
|  | **Grp4 Primary (n=23)** | 0.0083 |
|  | **Grp4 Recurrence (n=21)** | 0.0086 |
| ***B cells memory*** | **Grp4 All (n=44)** | 0.0004 |
|  | **Grp4 Primary (n=23)** | 0.0005 |
|  | **Grp4 Recurrence (n=21)** | 0.0002 |
| ***Plasma cells*** | **Grp4 All (n=44)** | 0.0003 |
|  | **Grp4 Primary (n=23)** | 0.0000 |
|  | **Grp4 Recurrence (n=21)** | 0.0006 |
| ***T cells CD8*** | **Grp4 All (n=44)** | 0.0399 |
|  | **Grp4 Primary (n=23)** | 0.0436 |
|  | **Grp4 Recurrence (n=21)** | 0.0358 |
| ***T cells CD4 naive*** | **Grp4 All (n=44)** | 0.0000 |
|  | **Grp4 Primary (n=23)** | 0.0000 |
|  | **Grp4 Recurrence (n=21)** | 0.0000 |
| ***T cells CD4 memory resting*** | **Grp4 All (n=44)** | 0.0260 |
|  | **Grp4 Primary (n=23)** | 0.0237 |
|  | **Grp4 Recurrence (n=21)** | 0.0286 |
| ***T cells CD4 memory activated*** | **Grp4 All (n=44)** | 0.0006 |
|  | **Grp4 Primary (n=23)** | 0.0005 |
|  | **Grp4 Recurrence (n=21)** | 0.0008 |
| ***T cells follicular helper*** | **Grp4 All (n=44)** | 0.0136 |
|  | **Grp4 Primary (n=23)** | 0.0136 |
|  | **Grp4 Recurrence (n=21)** | 0.0136 |
| ***T cells gamma delta*** | **Grp4 All (n=44)** | 0.0004 |
|  | **Grp4 Primary (n=23)** | 0.0004 |
|  | **Grp4 Recurrence (n=21)** | 0.0004 |
| ***T cells regulatory (Tregs)*** | **Grp4 All (n=44)** | 0.0074 |
|  | **Grp4 Primary (n=23)** | 0.0093 |
|  | **Grp4 Recurrence (n=21)** | 0.0053 |
| ***NK cells resting*** | **Grp4 All (n=44)** | 0.0062 |
|  | **Grp4 Primary (n=23)** | 0.0074 |
|  | **Grp4 Recurrence (n=21)** | 0.0048 |
| ***NK cells activated*** | **Grp4 All (n=44)** | 0.0010 |
|  | **Grp4 Primary (n=23)** | 0.0012 |
|  | **Grp4 Recurrence (n=21)** | 0.0008 |
| ***Monocytes*** | **Grp4 All (n=44)** | 0.0120 |
|  | **Grp4 Primary (n=23)** | 0.0125 |
|  | **Grp4 Recurrence (n=21)** | 0.0115 |
| ***Dendritic cells resting*** | **Grp4 All (n=44)** | 0.0006 |
|  | **Grp4 Primary (n=23)** | 0.0007 |
|  | **Grp4 Recurrence (n=21)** | 0.0005 |
| ***Dendritic cells activated*** | **Grp4 All (n=44)** | 0.0001 |
|  | **Grp4 Primary (n=23)** | 0.0001 |
|  | **Grp4 Recurrence (n=21)** | 0.0000 |
| ***Mast cells resting*** | **Grp4 All (n=44)** | 0.0027 |
|  | **Grp4 Primary (n=23)** | 0.0023 |
|  | **Grp4 Recurrence (n=21)** | 0.0031 |
| ***Mast cells activated*** | **Grp4 All (n=44)** | 0.0016 |
|  | **Grp4 Primary (n=23)** | 0.0016 |
|  | **Grp4 Recurrence (n=21)** | 0.0017 |
| ***Eosinophils*** | **Grp4 All (n=44)** | 0.0016 |
|  | **Grp4 Primary (n=23)** | 0.0013 |
|  | **Grp4 Recurrence (n=21)** | 0.0020 |
| ***Neutrophils*** | **Grp4 All (n=44)** | 0.0003 |
|  | **Grp4 Primary (n=23)** | 0.0003 |
|  | **Grp4 Recurrence (n=21)** | 0.0004 |
| ***Macrophage M0*** | **Grp4 All (n=44)** | 0.0025 |
|  | **Grp4 Primary (n=23)** | 0.0020 |
|  | **Grp4 Recurrence (n=21)** | 0.0031 |
| ***Macrophage M1*** | **Grp4 All (n=44)** | 0.0046 |
|  | **Grp4 Primary (n=23)** | 0.0033 |
|  | **Grp4 Recurrence (n=21)** | 0.0060 |
| ***Macrophage M2*** | **Grp4 All (n=44)** | 0.0109 |
|  | **Grp4 Primary (n=23)** | 0.0113 |
|  | **Grp4 Recurrence (n=21)** | 0.0104 |

**Supplementary Table 1A:** Tables displaying the mean and standard deviation values for each immune infiltrating cell type estimate for SHH, Group3 and Group4 patients for the entire study cohort, diagnosis only and recurrence only.

|  |  |  |  |
| --- | --- | --- | --- |
|  | **Cell Type** | **Group** | **Mean** |
|  | ***B cells*** | **SHH All (n=70)** | 0.0064 |
|  |  | **SHH Primary (n=33)** | 0.0063 |
|  |  | **SHH Recurrence (n=37)** | 0.0064 |
|  | ***T Cells*** | **SHH All (n=70)** | 0.0738 |
|  |  | **SHH Primary (n=33)** | 0.0666 |
|  |  | **SHH Recurrence (n=37)** | 0.0802 |
|  | ***NK Cells*** | **SHH All (n=70)** | 0.0129 |
|  |  | **SHH Primary (n=33)** | 0.0129 |
|  |  | **SHH Recurrence (n=37)** | 0.0128 |
|  | ***Myeloid Cels*** | **SHH All (n=70)** | 0.0526 |
|  |  | **SHH Primary (n=33)** | 0.0535 |
|  |  | **SHH Recurrence (n=37)** | 0.0517 |
|  | ***Plasma Cells*** | **SHH All (n=70)** | 0.0006 |
|  |  | **SHH Primary (n=33)** | 0.0003 |
|  |  | **SHH Recurrence (n=37)** | 0.0008 |

|  | **Cell Type** | **Group** | **Mean** |
| --- | --- | --- | --- |
|  | ***B cells*** | **Grp3 All (n=20)** | 0.0092 |
|  |  | **Grp3 Primary (n=6)** | 0.0102 |
|  |  | **Grp3 Recurrence (n=14)** | 0.0088 |
|  | ***T Cells*** | **Grp3 All (n=20)** | 0.1010 |
|  |  | **Grp3 Primary (n=6)** | 0.1028 |
|  |  | **Grp3 Recurrence (n=14)** | 0.1002 |
|  | ***NK Cells*** | **Grp3 All (n=20)** | 0.0045 |
|  |  | **Grp3 Primary (n=6)** | 0.0070 |
|  |  | **Grp3 Recurrence (n=14)** | 0.0035 |
|  | ***Myeloid Cels*** | **Grp3 All (n=20)** | 0.0466 |
|  |  | **Grp3 Primary (n=6)** | 0.0588 |
|  |  | **Grp3 Recurrence (n=14)** | 0.0413 |
|  | ***Plasma Cells*** | **Grp3 All (n=20)** | 0.0003 |
|  |  | **Grp3 Primary (n=6)** | 0.0001 |
|  |  | **Grp3 Recurrence (n=14)** | 0.0004 |

| **Cell Type** | **Group** | **Mean** |  |  |  |
| --- | --- | --- | --- | --- | --- |
| ***B cells*** | **Grp4 All (n=44)** | 0.0088 |  |  |  |
|  | **Grp4 Primary (n=23)** | 0.0088 |  |  |  |
|  | **Grp4 Recurrence (n=21)** | 0.0088 |  |  |  |
| ***T Cells*** | **Grp4 All (n=44)** | 0.0879 |  |  |  |
|  | **Grp4 Primary (n=23)** | 0.0910 |  |  |  |
|  | **Grp4 Recurrence (n=21)** | 0.0844 |  |  |  |
| ***NK Cells*** | **Grp4 All (n=44)** | 0.0072 |  |  |  |
|  | **Grp4 Primary (n=23)** | 0.0087 |  |  |  |
|  | **Grp4 Recurrence (n=21)** | 0.0056 |  |  |  |
| ***Myeloid Cels*** | **Grp4 All (n=44)** | 0.0370 |  |  |  |
|  | **Grp4 Primary (n=23)** | 0.0355 |  |  |  |
|  | **Grp4 Recurrence (n=21)** | 0.0387 |  |  |  |
| ***Plasma Cells*** | **Grp4 All (n=44)** | 0.0003 |  |  |  |
|  | **Grp4 Primary (n=23)** | 0.0000 |  |  |  |
|  | **Grp4 Recurrence (n=21)** | 0.0006 |  |  |  |
| **Supplementary Table 1B:** Tables displaying the mean and standard deviation values for each immune infiltrating cell class estimate for SHH, Group3 and Group4 patients for the entire study cohort, diagnosis only and recurrence only. | | | | |  |
|  |  |  |  |  |  |

| **Between Groups - Combined cohort** | | | | | |  |
| --- | --- | --- | --- | --- | --- | --- |
|  |  |  |  |  |  |  |
| **Cell_Type** | **Comparison** | **Z** | **P.unadj** | **P.adj** | **Result** |  |
| ***B Cells*** | **Group 3 - Group 4** | 0.751 | 0.453 | 1.000 | *No significant difference* |  |
|  | **Group 3 - SHH** | 2.848 | 0.004 | 0.013 | ***Group 3 (0.009) higher than SHH (0.006)*** |  |
|  | **Group 4 - SHH** | 2.700 | 0.007 | 0.021 | ***Group 4 (0.009) higher than SHH (0.006)*** |  |
| ***T Cells*** | **Group 3 - Group 4** | 1.065 | 0.287 | 0.860 | *No significant difference* |  |
|  | **Group 3 - SHH** | 3.021 | 0.003 | 0.008 | ***Group 3 (0.101) higher than SHH (0.074)*** |  |
|  | **Group 4 - SHH** | 2.488 | 0.013 | 0.038 | ***Group 4 (0.088) higher than SHH (0.074)*** |  |
| ***NK Cells*** | **Group 3 - Group 4** | -1.523 | 0.128 | 0.384 | *No significant difference* |  |
|  | **Group 3 - SHH** | -4.228 | 0.000 | 0.000 | ***Group 3 (0.005) lower than SHH (0.013)*** |  |
|  | **Group 4 - SHH** | -3.438 | 0.001 | 0.002 | ***Group 4 (0.007) lower than SHH (0.013)*** |  |
| ***Myeloid Cells*** | **Group 3 - Group 4** | 1.533 | 0.125 | 0.376 | *No significant difference* |  |
|  | **Group 3 - SHH** | -1.103 | 0.270 | 0.810 | *No significant difference* |  |
|  | **Group 4 - SHH** | -3.603 | 0.000 | 0.001 | ***Group 4 (0.037) lower than SHH (0.053)*** |  |
| ***Plasma Cells*** | **Group 3 - Group 4** | 0.266 | 0.791 | 1.000 | *No significant difference* |  |
|  | **Group 3 - SHH** | -1.123 | 0.261 | 0.784 | *No significant difference* |  |
|  | **Group 4 - SHH** | -1.853 | 0.064 | 0.192 | *No significant difference* |  |

| **Between Groups - Primary only** | | | | | |  |
| --- | --- | --- | --- | --- | --- | --- |
|  |  |  |  |  |  |  |
| **Cell_Type** | **Comparison** | **Z** | **P.unadj** | **P.adj** | **Result** |  |
| ***B Cells*** | **Group 3 - Group 4** | 0.445 | 0.656 | 1.000 | *No significant difference* |  |
|  | **Group 3 - SHH** | 1.752 | 0.080 | 0.239 | *No significant difference* |  |
|  | **Group 4 - SHH** | 2.112 | 0.035 | 0.104 | *No significant difference* |  |
| ***T Cells*** | **Group 3 - Group 4** | 0.562 | 0.574 | 1.000 | *No significant difference* |  |
|  | **Group 3 - SHH** | 2.059 | 0.040 | 0.119 | *No significant difference* |  |
|  | **Group 4 - SHH** | 2.415 | 0.016 | 0.047 | ***Group 4 (0.091) higher than SHH (0.067)*** |  |
| ***NK Cells*** | **Group 3 - Group 4** | -0.632 | 0.527 | 1.000 | *No significant difference* |  |
|  | **Group 3 - SHH** | -1.833 | 0.067 | 0.200 | *No significant difference* |  |
|  | **Group 4 - SHH** | -1.928 | 0.054 | 0.162 | *No significant difference* |  |
| ***Myeloid Cells*** | **Group 3 - Group 4** | 1.681 | 0.093 | 0.278 | *No significant difference* |  |
|  | **Group 3 - SHH** | 0.119 | 0.905 | 1.000 | *No significant difference* |  |
|  | **Group 4 - SHH** | -2.643 | 0.008 | 0.025 | ***Group 4 (0.036) lower than SHH (0.053)*** |  |
| ***Plasma Cells*** | **Group 3 - Group 4** | 1.064 | 0.288 | 0.863 | *No significant difference* |  |
|  | **Group 3 - SHH** | -0.196 | 0.844 | 1.000 | *No significant difference* |  |
|  | **Group 4 - SHH** | -2.116 | 0.034 | 0.103 | *No significant difference* |  |

| **Between Groups - Recurrent only** | | | | | |  |
| --- | --- | --- | --- | --- | --- | --- |
|  |  |  |  |  |  |  |
| **Cell_Type** | **Comparison** | **Z** | **P.unadj** | **P.adj** | **Result** |  |
| ***B Cells*** | **Group 3 - Group 4** | 0.536 | 0.592 | 1.000 | *No significant difference* |  |
|  | **Group 3 - SHH** | 2.131 | 0.033 | 0.099 | *No significant difference* |  |
|  | **Group 4 - SHH** | 1.771 | 0.077 | 0.230 | *No significant difference* |  |
| ***T Cells*** | **Group 3 - Group 4** | 1.085 | 0.278 | 0.834 | *No significant difference* |  |
|  | **Group 3 - SHH** | 1.863 | 0.062 | 0.187 | *No significant difference* |  |
|  | **Group 4 - SHH** | 0.770 | 0.442 | 1.000 | *No significant difference* |  |
| ***NK Cells*** | **Group 3 - Group 4** | -1.019 | 0.308 | 0.925 | *No significant difference* |  |
|  | **Group 3 - SHH** | -3.674 | 0.000 | 0.001 | ***Group 3 (0.003) lower than SHH (0.013)*** |  |
|  | **Group 4 - SHH** | -2.933 | 0.003 | 0.010 | ***Group 4 (0.006) lower than SHH (0.013)*** |  |
| ***Myeloid Cells*** | **Group 3 - Group 4** | 0.580 | 0.562 | 1.000 | *No significant difference* |  |
|  | **Group 3 - SHH** | -1.484 | 0.138 | 0.413 | *No significant difference* |  |
|  | **Group 4 - SHH** | -2.437 | 0.015 | 0.044 | ***Group 4 (0.039) lower than SHH (0.052)*** |  |
| ***Plasma Cells*** | **Group 3 - Group 4** | -0.551 | 0.582 | 1.000 | *No significant difference* |  |
|  | **Group 3 - SHH** | -1.172 | 0.241 | 0.723 | *No significant difference* |  |
|  | **Group 4 - SHH** | -0.650 | 0.515 | 1.000 | *No significant difference* |  |

**Supplementary Table 1C:** Adjusted p-values for each immune infiltrating cell class for each molecular group vs others, derived from Dunns multiple comparison tests.
