# Supplementary material for: MIF-CD74 signaling drives immune modulation in medulloblastoma
Source: Neuro Oncol. 2026 Feb 6;28(5):1316–34. doi: 10.1093/neuonc/noag020 (PMC13186457; doi:10.1093/neuonc/noag020)
Supplement: noag020_Supplementary_Data [file noag020_supplementary_data.zip › Supplementary table 2.docx]

| **Cohort** | **Okonechnikov et al** | **Newcastle** | **GOSH** | **Lisbon** |
| --- | --- | --- | --- | --- |
| **No. of samples** | **86** | **54** | **6** | **6** |
| **Analysis** | **RNA-seq** | **RNA-seq** | **Spatial Phenotypic Analysis** | **Spatial Phenotypic Analysis** |
| Gender |  |  |  |  |
| Male | 58 | 27 | 0 | 4 |
| Female | 28 | 25 | 0 | 1 |
| Unknown | 0 | 2 | 6 | 1 |
| Age at diagnosis |  |  |  |  |
| Infant (0-3 years) | 5 | 4 | 3 | 0 |
| Child (3-18 years) | 24 | 16 | 3 | 3 |
| Adult (18+) | 14 | 0 | 0 | 2 |
| Unknown | 0 | 0 | 0 | 1 |
| Sample status |  |  |  |  |
| Primary | 43 | 20 | 6 | 6 |
| Recurrence | 43 | 34 | 6 | 6 |
| Paired | 43 | 14 | 6 | 6 |
| Metastatic stage at diagnosis |  |  |  |  |
| M0/1 | 33 | 15 | 0 | 4 |
| M2/3 | 10 | 5 | 0 | 1 |
| Unknown | 0 | 0 | 6 | 1 |
| Histology at diagnosis |  |  |  |  |
| Classic | 12 | 10 | 2 | 0 |
| Demoplastic/Nodular | 20 | 1 | 0 | 2 |
| LCA | 11 | 1 | 4 | 0 |
| Unknown | 0 | 8 | 0 | 4 |
| Molecular group at diagnosis^1^ |  |  |  |  |
| WNT | 0 | 1 | 0 | 0 |
| SHH | 24 | 9 | 1 | 3 |
| Group 3 | 6 | 1 | 3 | 0 |
| Group 4 | 13 | 9 | 1 | 3 |
| Group 3/4 | 0 | 0 | 1 | 0 |
| Unknown | 0 | 0 | 0 | 0 |
| Upfront treatment received |  |  |  |  |
| Surgery, radiotherapy & chemotherapy | 38 | 11 | 4 | 5 |
| Surgery & chemotherapy | 5 | 1 | 2 | 0 |
| Unknown | 0 | 8 | 0 | 1 |
| ¹ Molecular Group was determined using the "MNP brain tumour classifier v12.8" (Capper et al., 2018). | | | |  |

**Supplementary Table 2:** **Cohort Demographics and Clinical Characteristics**. Summary of the clinical, molecular, and therapeutic features of the medulloblastoma sample cohorts included in this study. ¹Molecular Group was determined using the "MNP brain tumour classifier v12.8" (Capper et al., 2018).
