# Supplementary figures and images for: MIF-CD74 signaling drives immune modulation in medulloblastoma
Source: Neuro Oncol. 2026 Feb 6;28(5):1316–34. doi: 10.1093/neuonc/noag020 (PMC13186457; doi:10.1093/neuonc/noag020)

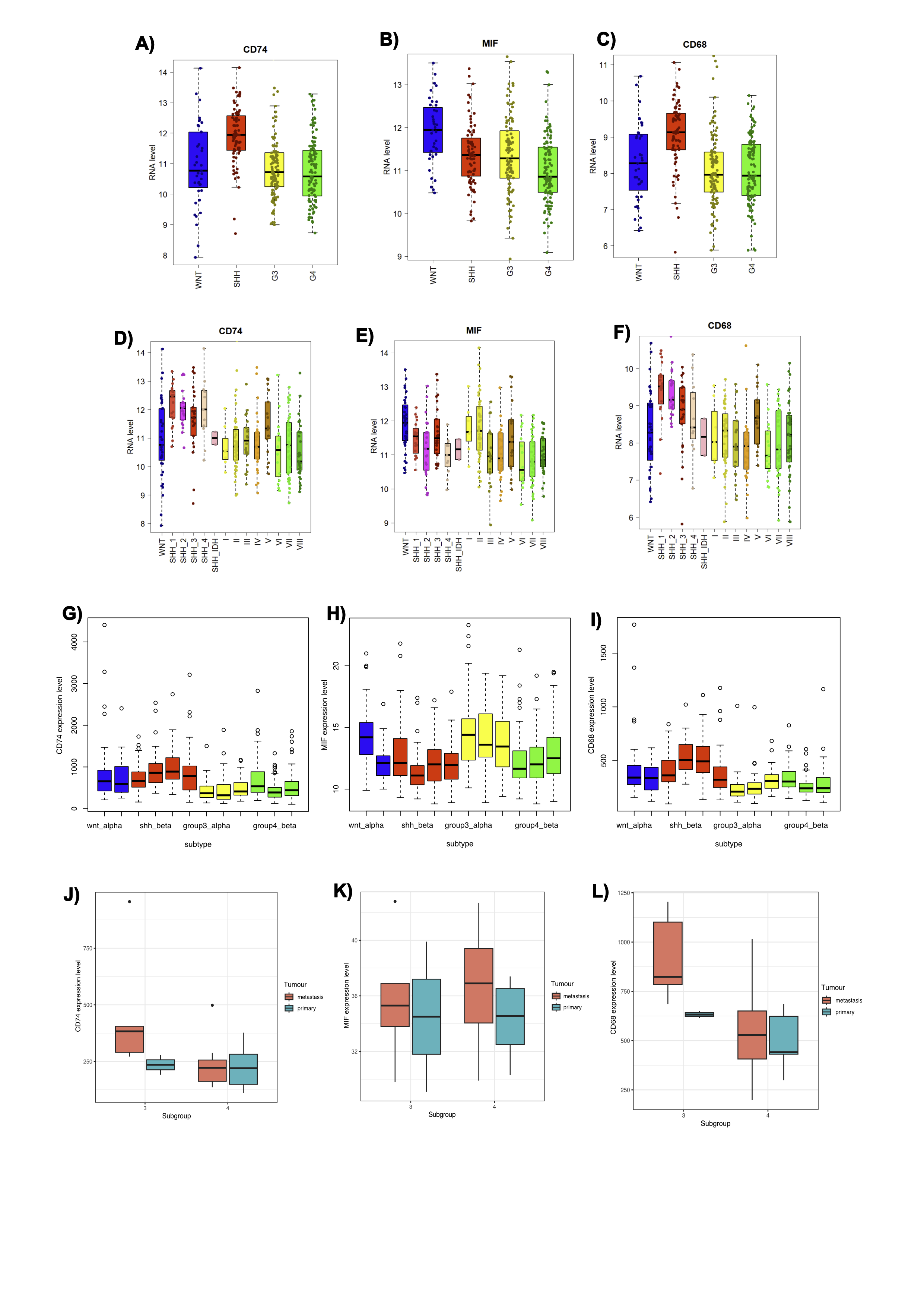

Supplement: noag020_Supplementary_Data [file noag020_supplementary_data.zip › SupplementalFigure4_1.tiff]

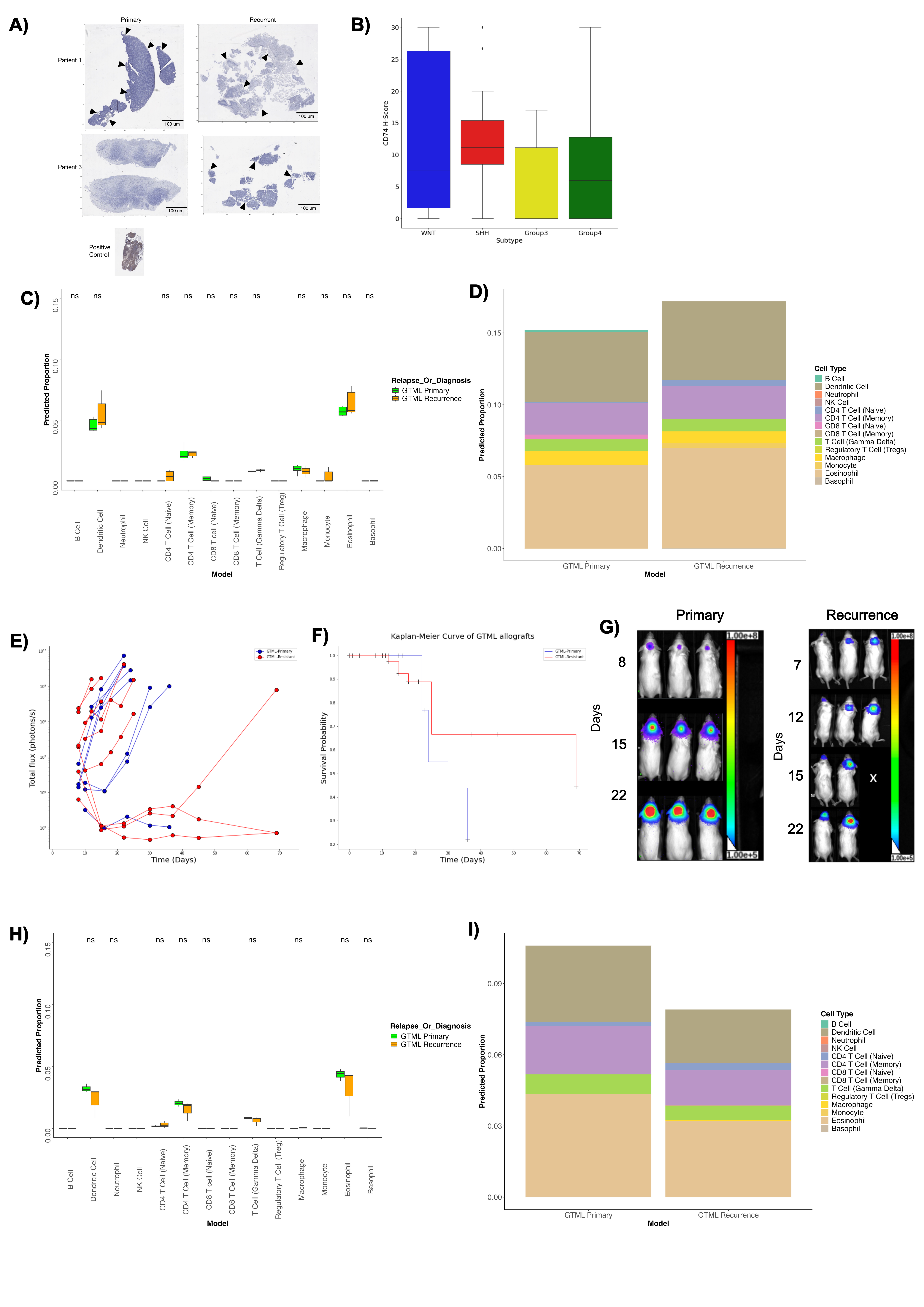

Supplement: noag020_Supplementary_Data [file noag020_supplementary_data.zip › SupplementalFigure5(4)_new.tiff]

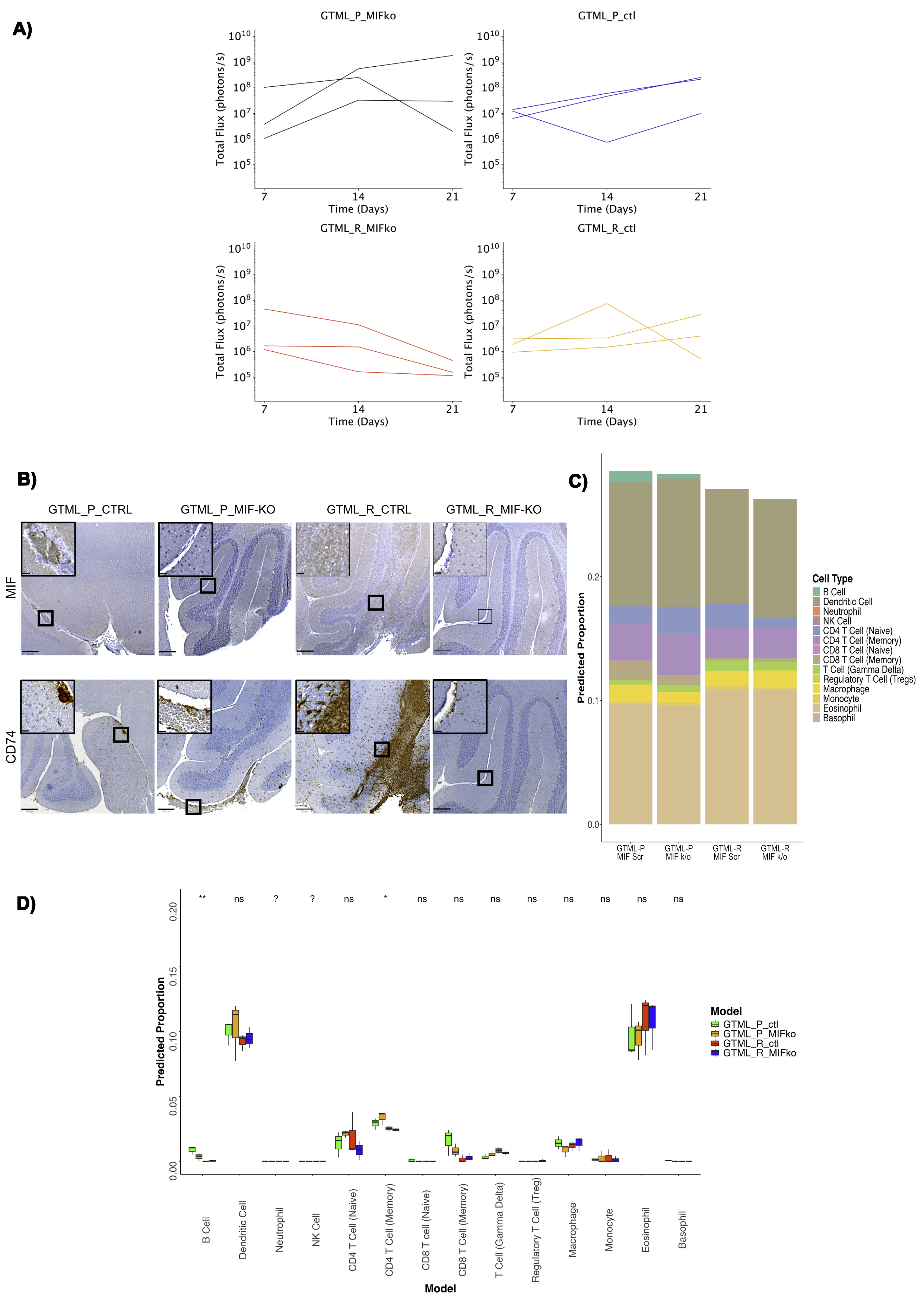

Supplement: noag020_Supplementary_Data [file noag020_supplementary_data.zip › SupplementalFigure6_1.tiff]

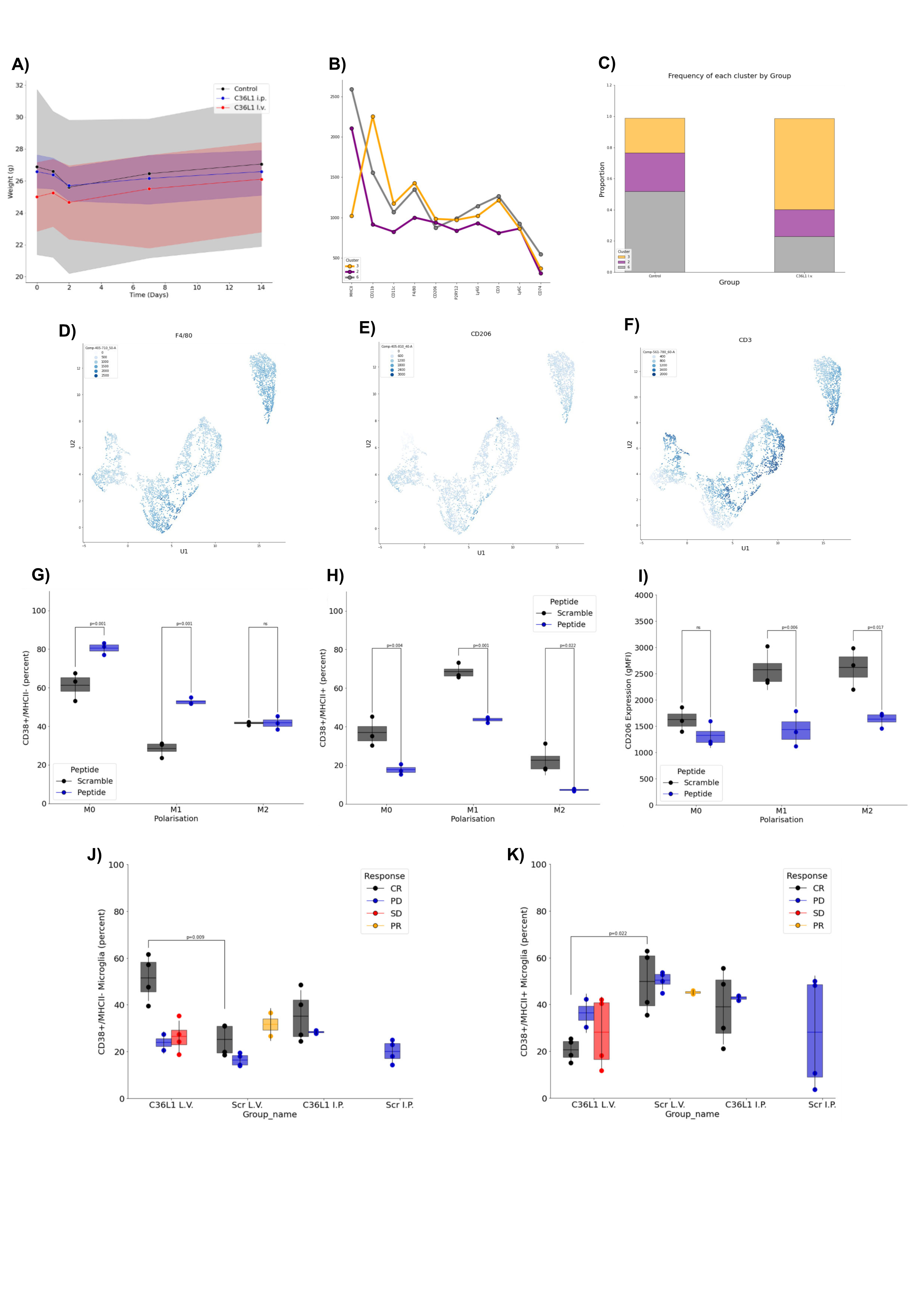

Supplement: noag020_Supplementary_Data [file noag020_supplementary_data.zip › SupplementalFigure7_1.tiff]

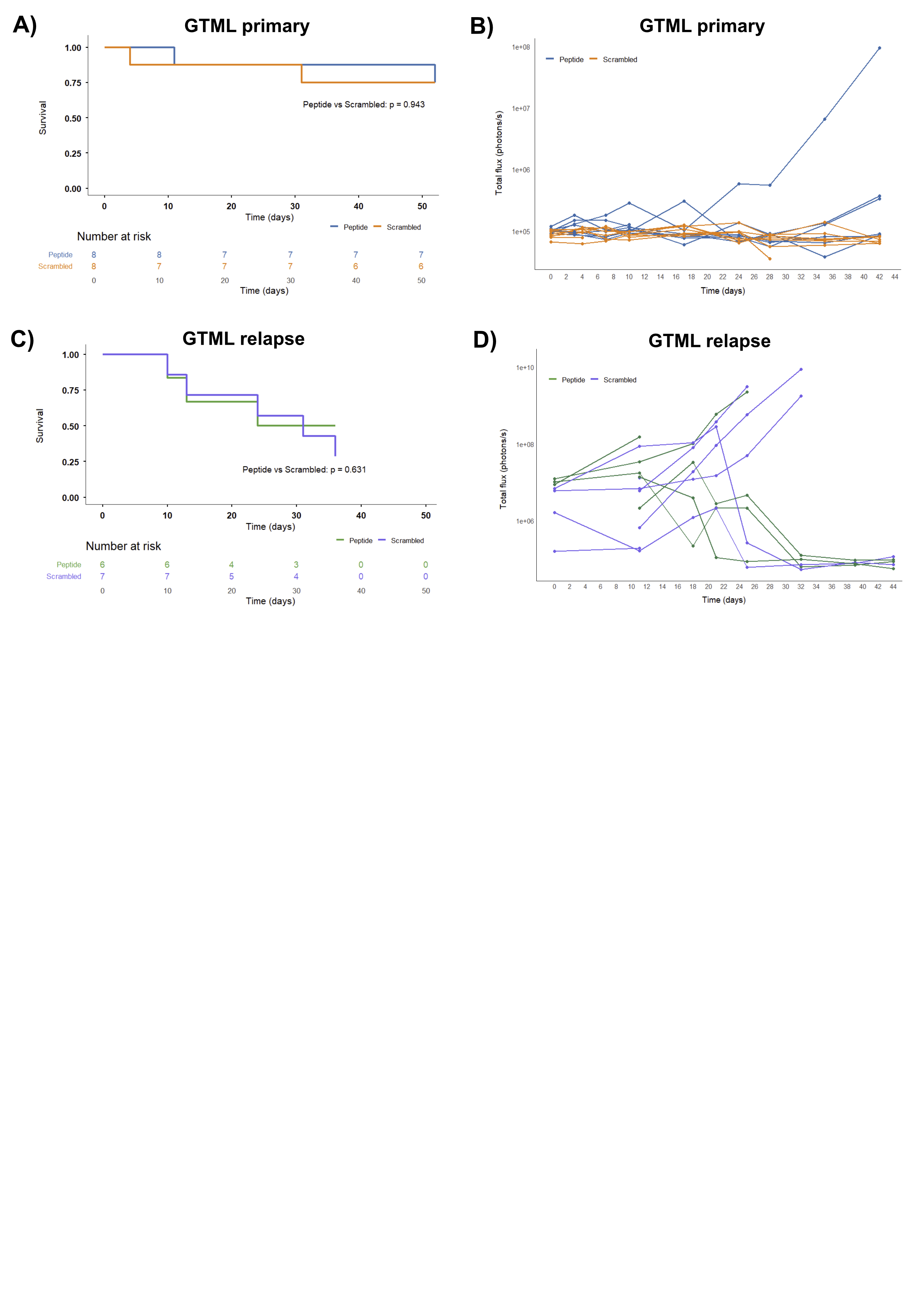

Supplement: noag020_Supplementary_Data [file noag020_supplementary_data.zip › SupplementalFigure8_1.tiff]

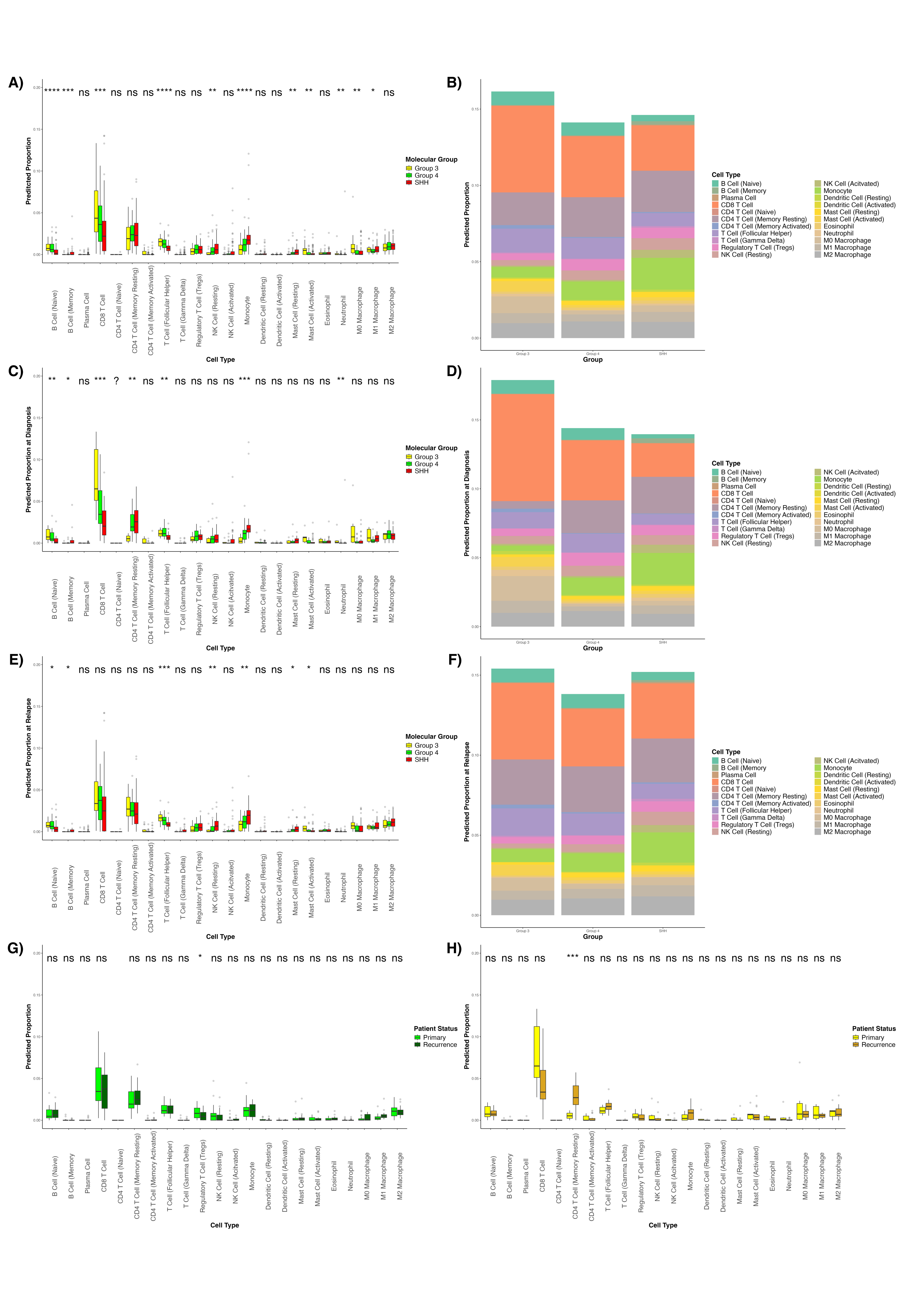

Supplement: noag020_Supplementary_Data [file noag020_supplementary_data.zip › SupplementalFigure1_1.tiff]

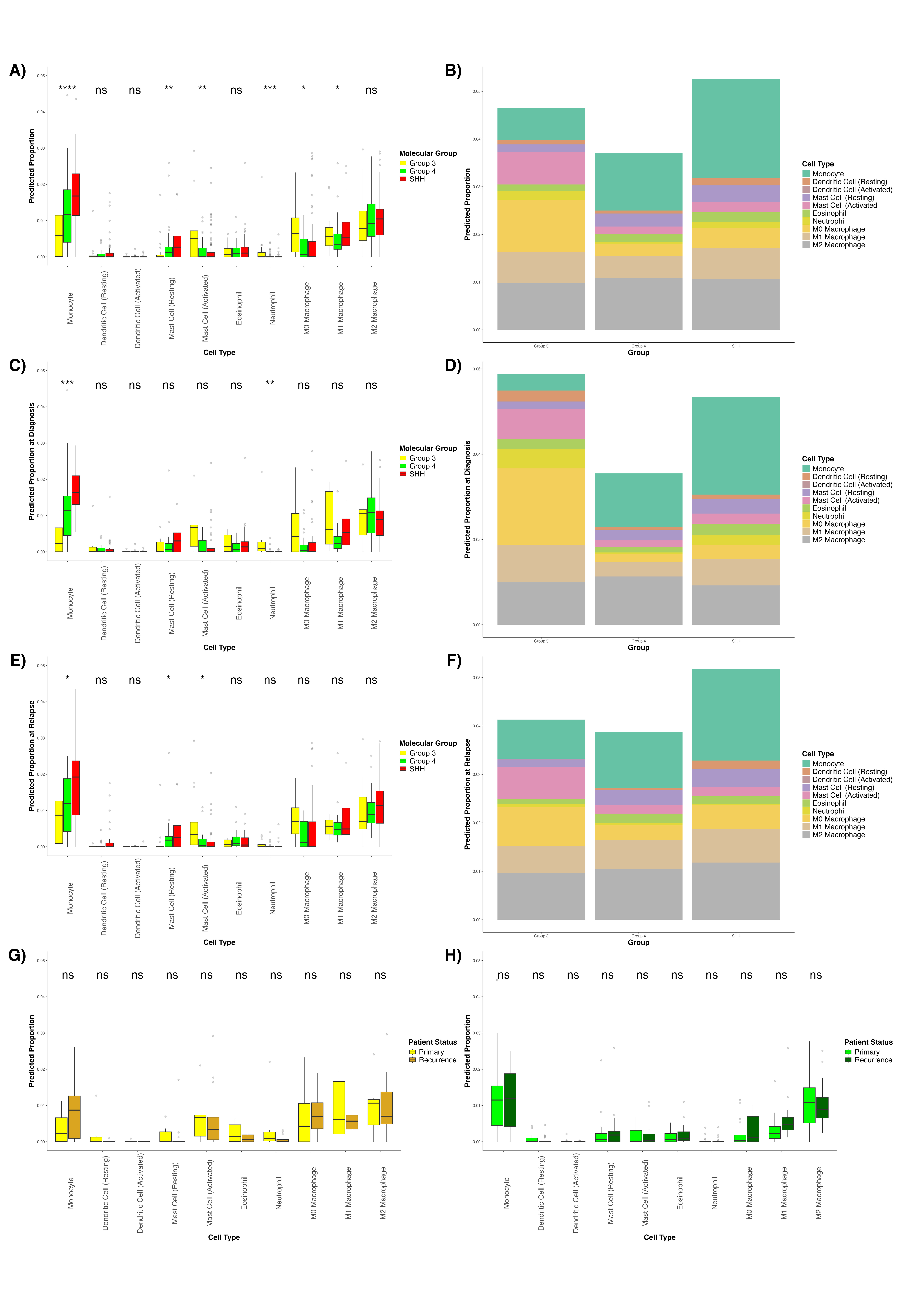

Supplement: noag020_Supplementary_Data [file noag020_supplementary_data.zip › SupplementalFigure2_1.tiff]

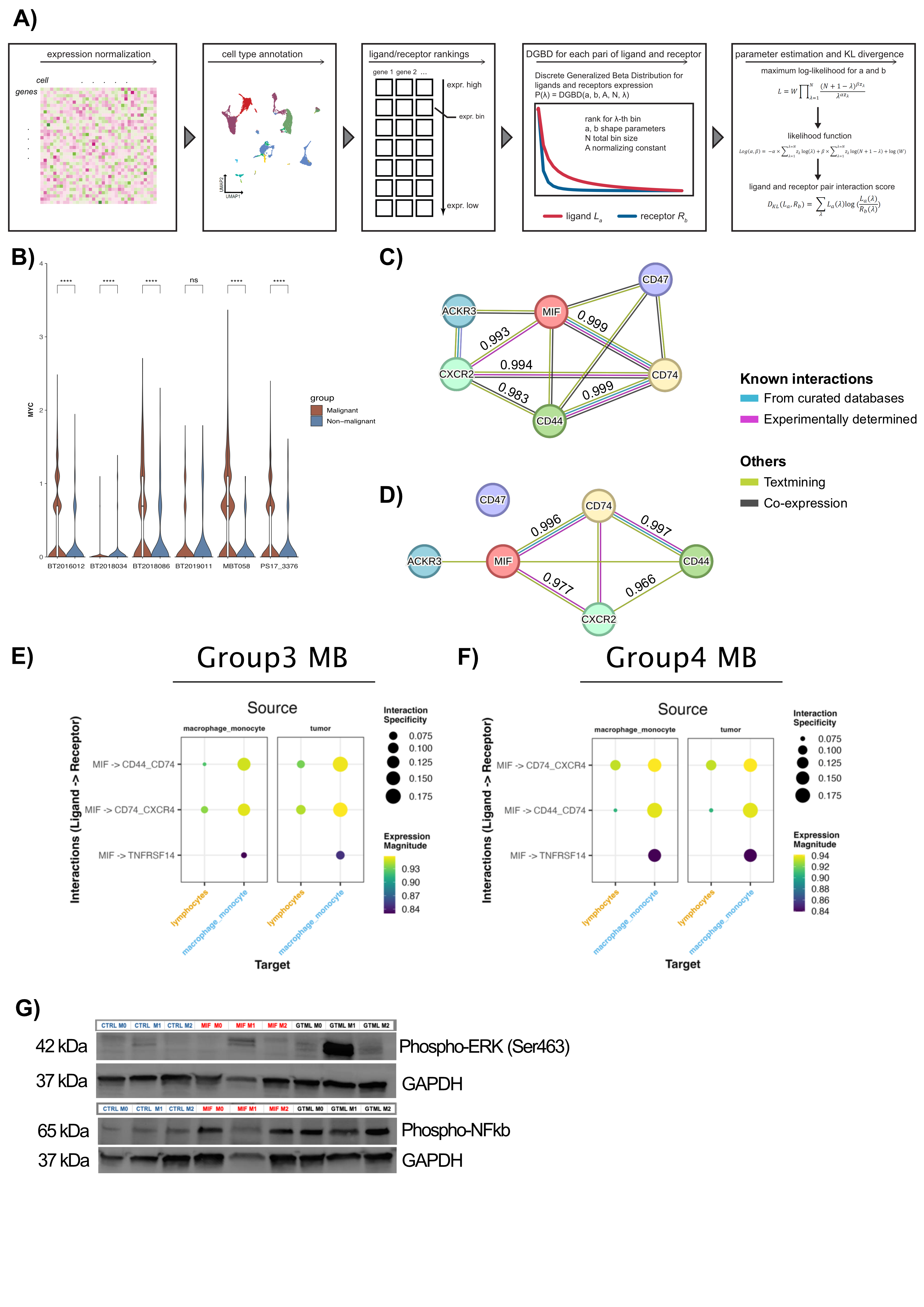

Supplement: noag020_Supplementary_Data [file noag020_supplementary_data.zip › SupplementalFigure3_amended.tiff]
